# Conserved long-range interactions are required for stable folding of orthoflaviviral genomic RNA

**DOI:** 10.1093/nar/gkaf514

**Published:** 2025-06-16

**Authors:** Michael Z Palo, Betty Ha, Christopher P Lapointe, Carlos Alvarado, John Janetzko, Jan E Carette, Joseph D Puglisi, Elisabetta Viani Puglisi

**Affiliations:** Department of Structural Biology, Stanford University School of Medicine, Stanford, CA 94305, United States; Department of Structural Biology, Stanford University School of Medicine, Stanford, CA 94305, United States; Department of Molecular and Cellular Physiology, Stanford University School of Medicine, Stanford, CA 94305, United States; Department of Structural Biology, Stanford University School of Medicine, Stanford, CA 94305, United States; Department of Structural Biology, Stanford University School of Medicine, Stanford, CA 94305, United States; Department of Molecular and Cellular Physiology, Stanford University School of Medicine, Stanford, CA 94305, United States; Department of Microbiology and Immunology, Stanford University School of Medicine, Stanford, CA 94305, United States; Department of Structural Biology, Stanford University School of Medicine, Stanford, CA 94305, United States; Department of Structural Biology, Stanford University School of Medicine, Stanford, CA 94305, United States

## Abstract

Long-range tertiary interactions are a widespread structural feature in viral RNAs (vRNAs) and mRNAs. In the orthoflaviviruses, conserved complementary sequences in the 5′ and 3′ terminal regions have an essential role in viral replication. Long-range pairing of these conserved sequences is proposed to facilitate a switch between two alternative vRNA conformations. Yet the detailed nature of these interactions, their relative populations and their exchange are required to formulate a mechanistic model of their role in regulation of the viral life cycle. Here, we used single-molecule Förster resonance energy transfer to study the global conformation of vRNAs by measuring their end-to-end distances. We observed that vRNA conformation is heterogeneous, and that conformers with close end-to-end distances have unusual kinetic stability when compared with mRNA lacking these specific long-range interactions. vRNAs also partition between at least two stable states with a large rearrangement of the terminal regions (>50 Å change in end-to-end distance). We demonstrate that this bistability depends on long-range interactions and is modulated by host factors such as the initiation factor complex eIF4F. Understanding how vRNA and its stability is influenced by interactions with other host and viral factors will help to elucidate a mechanistic role for these highly conserved orthoflaviviral sequences.

## Graphical Abstract

**Graphical Abstract:**
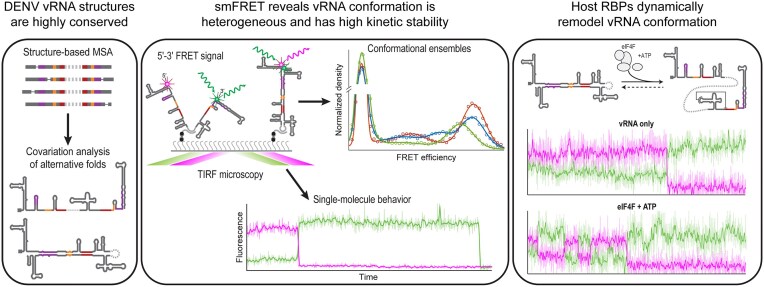

## Introduction

Orthoflaviviruses are a genus of enveloped, positive sense single-stranded RNA (ssRNA) viruses that pose current and emerging public health threats [1]. Important human pathogens like dengue virus (DENV), West Nile virus (WNV), and Zika virus (ZIKV) circulate between mammalian or avian hosts and insect vectors [2] and make this group the leading cause of insect-borne viral disease in the world. However, vaccines have been developed for only a few species, and there are no approved antiviral drugs to combat infection. A deeper understanding of mechanisms of orthoflavivirus replication is needed to counter the current and emerging threats of these viruses.

**Figure 1.**
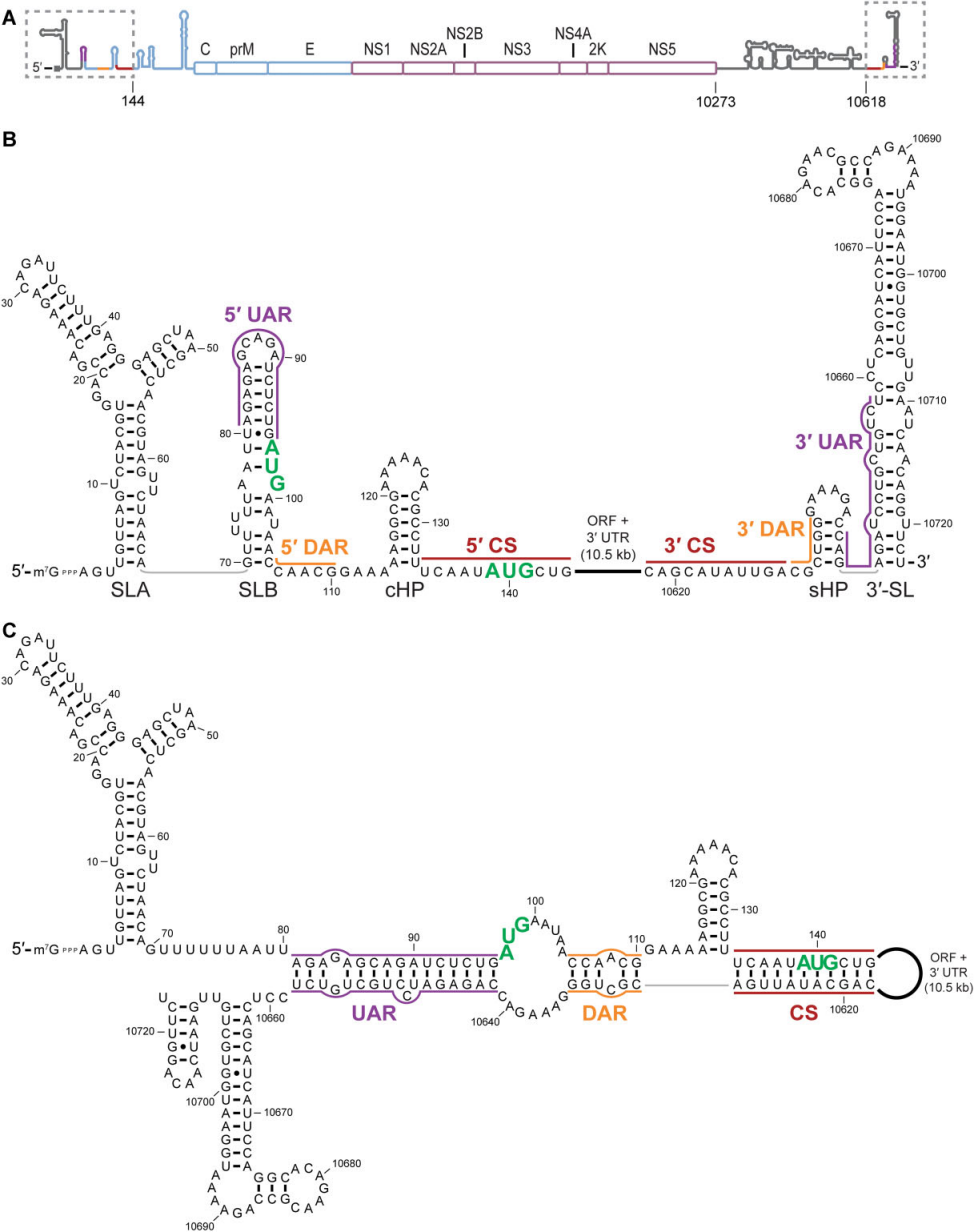
Orthoflaviviral RNA can fold into alternative secondary structures. (**A**) Organization of orthoflavivirus vRNA. The vRNA polyprotein ORF is subdivided into the protein products produced by proteolytic cleavage during infection. Conserved terminal structures are shown as cartoons, structural proteins as blue boxes, and nonstructural proteins as purple boxes. Numbering corresponds to a model orthoflavivirus, DENV2 strain 16681. Regions shown in panels (B) and (C) are boxed. (**B**) Proposed secondary structure of the terminal regions of DENV2 strain 16681, in the extended conformation. A schematic of vRNA organization is shown at the top, with conserved terminal structures shown as cartoons, structural protein genes as blue boxes, and nonstructural protein genes as purple boxes. Start codons for the viral ORF are highlighted in bold green font, and conserved complementary sequences are annotated with purple, yellow, and maroon lines. Named secondary structure elements are labeled below each stem. (**C**) Proposed secondary structure of the cyclized conformation of DENV2 strain 16681. Annotations are the same as in panel (B).

Orthoflaviviral vRNAs are 10–11 kb in length and have a 5′-7-methylguanosine (m^7^G) cap structure but lack a poly (A) tail. They contain a long open reading frame (ORF) encoding a polyprotein that is co- and post-translationally processed into three structural and seven nonstructural proteins (Fig. 1A). The 5′ and 3′ terminal regions feature evolutionarily conserved sequence and structural elements that regulate the viral life cycle (Fig. 1B). Most prominently, the 5′ stem-loop A (SLA) and 3′ stem loop (3′-SL) are conserved among all orthoflaviviruses (Fig. 1B) [3], and 2–3 pairs of 5–25 nt long complementary sequences are present near the 5′ and 3′ ends of all but the insect-specific flaviviruses [4–6].

SLA is a promoter element recognized by the viral RNA polymerase NS5 for initiation of negative strand viral RNA (vRNA) synthesis, the first step of vRNA replication [7, 8]. The complementary sequences UAR, DAR, and CS (upstream AUG region, downstream AUG region, and cyclization sequence, respectively, in most mosquito-borne flaviviruses) are proposed to noncovalently cyclize vRNA by long-range base pairing [9–19] and mediate intramolecular activation of *de novo* replication initiation at the 3′ end by 5′ SLA-bound NS5 [7, 8]. Complementarity of these sequences is essential for vRNA replication but not initial translation [17–22]. The UAR also base pairs with nearby UTR sequences to form the conserved local structures stem-loop B (SLB) and 3′-SL (Fig. 1B), which are incompatible with long-range interactions (Fig. 1C) [19, 23–25]. These alternative conformations of the vRNA terminal regions are proposed to function in different stages of infection, with the extended conformation favoring translation by host ribosomes, and the cyclized conformation facilitating genome replication by viral factors [19, 26].

Intramolecular proximity of the 5′ and 3′ ends of RNA has been implicated in other essential biological processes like translation initiation and RNA decay [27]. Whereas proximity could be driven by protein–protein interactions on a given RNA, theoretical, and experimental results have demonstrated that an end-to-end distance of ∼5 nm is a sequence and length-independent feature of natural RNAs, mediated by global compaction of RNA through folding of secondary structure elements [28–31]. Importantly, this 5′-3′ proximity is achieved without extended long-range stems like those found in orthoflavivirus vRNAs. Placement of SLA at the 5′ end of unrelated RNA sequences lacking 5′-3′ complementary sequences and 3′-SL still activates negative strand RNA synthesis *in vitro* [7], suggesting that the intrinsic 5′-3′ proximity of RNA can facilitate replication initiation. Nevertheless, inclusion of complementary sequences enhanced the yield of newly synthesized RNA by 3- to 6-fold on these templates. Understanding the mechanism by which complementary sequences enhance replication initiation requires a detailed understanding of vRNA conformation.

Orthoflaviviral vRNA structure has been inferred from bioinformatic analysis [4, 5, 10], sequence determinants of viral replication [10, 12, 14, 15, 20, 32], and biochemical characterization by chemical probing [24, 33–38]. Analysis of the interaction between the 5′ and 3′ terminal regions as separate transcripts *in trans* or linked as a minigenome construct has shown a dependence of RNA conformation on the conserved complementary sequences [14, 18, 26, 39]. However, these studies have potentially perturbed the conformational ensemble of vRNA by transforming an intramolecular interaction to a bimolecular one. vRNA fragments also remove nearly the entire ∼10 kb coding region which contributes to global vRNA folding [35, 40]. In-cell and *in vitro* SHAPE probing of full-length DENV, WNV, and ZIKV vRNAs has suggested that the extended conformation is favored in the cytoplasm, while the cyclized conformation is stabilized in virions [35–38, 41]. The conformation of protein-free vRNA *in vitro* varied between strains. Ensemble averaging of nucleotide chemical accessibility in these methods may hide transient interactions and minor but functionally important states. Biophysical experiments to characterize single vRNA molecules using atomic force microscopy also relied on constraining much of the RNA to be double-stranded by annealing a long antisense RNA [7, 14], potentially biasing vRNA towards long-range interactions.

Determining a mechanistic role for complementary sequences in the orthoflavivirus life cycle requires methods to measure their interaction and track vRNA conformation during translation and vRNA replication. Single-molecule Förster resonance energy transfer (smFRET) is a powerful tool for measuring the conformations of biomolecules, especially for assessing heterogeneity in an ensemble and observing dynamic changes in conformation over time. Previous measurements of the end-to-end distance of messenger RNAs (mRNAs) by smFRET between fluorescent dye-labeled 5′ and 3′ ends revealed that mRNA conformation is dynamic, transitioning between multiple states with discrete end-to-end distances [30]. It is unknown if vRNA conformation is similarly heterogeneous and able to interconvert spontaneously between alternative structures that interact differently with host and viral factors to regulate the viral life cycle, especially during the core steps of translation and vRNA replication.

Here, we use smFRET to probe the orthoflavivirus vRNA conformational landscape by measuring the end-to-end distance and assess the role of conserved complementary sequences in establishing vRNA structure. We find that vRNA minigenome constructs fold into both extended and cyclized states in the absence of proteins that are conserved across diverse orthoflaviviruses. Both conformations are unusually kinetically stable relative to those of other natural RNAs, and this stability depends on multipartite long-range interactions. We observe similar conformational heterogeneity in full-length DENV vRNA, suggesting that the equilibrium between extended and cyclized is finely tuned. Finally, we show that eukaryotic initiation factors 4F (eIF4F) and 4B (eIF4B) destabilize the cyclized vRNA conformation in an ATP-dependent process. eIF4F and other viral and host factors may interact with vRNA during infection to trigger the proposed regulatory switch between alternative vRNA conformations.

## Materials and methods

### Bioinformatic analysis of DENV terminal regions

DENV vRNA sequences (taxid:12 637) were downloaded from NCBI Virus (https://www.ncbi.nlm.nih.gov/labs/virus/vssi/#/) with the “Nucleotide Completeness: complete” filter applied (4127 sequences). Sequences were aligned using Clustal Omega 1.2.4 [42] with default settings to generate an initial multiple sequence alignment (MSA) for visual verification of sequence completeness using Jalview [43]. Identical sequences were removed so that only one copy was present in the MSA. We frequently observed that vRNA sequences annotated as “complete” contained only the complete ORF with absent or partial UTRs. Sequences lacking the conserved 5′-AG terminal dinucleotide and predicted SLA or 5′-CU terminal dinucleotide and predicted 3′-SL were manually removed from the MSA. We also removed sequences with long, perfectly complementary inverted repeats near the terminal hairpins, which could have originated via a fold-back mechanism during reverse transcription [4]. The remaining sequences (1418 sequences) were trimmed to the terminal regions (5′-UTR and capsid coding region structures; 3′-UTR) and realigned using LaRA2 [44]. RNAalifold [45] was used to predict consensus secondary structures for the individual UTRs in the extended conformation. The consensus secondary structure of the cyclized conformation was manually annotated based on previous studies. Sequence conservation and base pair covariation were analyzed and visualized with R2R 1.0.7 [46] using default settings. Secondary structure diagrams were stylistically edited in Adobe Illustrator to produce final versions displayed in figures.

### Preparation of *in vitro* transcription templates

All oligonucleotides were purchased from Integrated DNA Technologies. Double-stranded DNA (dsDNA) fragments encoding the orthoflaviviral 5′-3′ UTR, NanoLuc, and human β-globin mRNA (NM_000518.5) sequences downstream of a T7 promoter were purchased from Integrated DNA Technologies. β-globin mRNA transcription from the T7 φ6.5 promoter (TAATACGACTCACTATA) was facilitated by addition of a single guanosine to the 5′ end of the sequence. Orthoflaviviral sequences were transcribed from the T7 φ2.5 promoter (TAATACGACTCACTATT) using the native 5′-AG as the transcription start site. dsDNA fragments encoding orthoflaviviral sequences were cloned into pUC19 using the NEBuilder HiFi DNA Assembly Master Mix (NEB, E2621S). Cyc+, UAR rescue 1, and UAR rescue 2 sequences were cloned from the WT DENV2 5′-3′ UTR, DENV2 UAR mutant 1 5′-3′ UTR, and DENV2 UAR mutant 2 5′-3′ UTR plasmids, respectively, using site-directed mutagenesis with the QuikChange Lightning Site-Directed Mutagenesis Kit (Agilent Technologies, 210519). Disruption and reconstitution of long-range interactions by these substitutions was computationally evaluated using the partition program in RNAstructure (version 6.5) [47]. Plasmids were purified from DH5α *Escherichia coli* (Invitrogen, 18265017) with HiSpeed Plasmid Midi (Qiagen, 12643) or Maxi kits (Qiagen, 12663), linearized using XbaI restriction enzyme (NEB, R0145L), and purified by phenol–chloroform extraction and ethanol precipitation. Linear DNA template encoding human β-globin with a poly (A)_30_ tail was amplified by polymerase chain reaction as described previously [48].

Plasmids pD2/IC-30P-A and pDVRep encoding the full-length DENV2 strain 16681 strain cDNA and RLuc replicons, respectively, downstream of T7 promoters were described previously [49]. To ensure production of noninfectious vRNA, the catalytic GDD motif in NS5 (polyprotein residues 3153–3155) was mutated to GAA using the QuikChange Lightning Site-Directed Mutagenesis kit. Propagation of plasmids encoding vRNA constructs was performed in One Shot Stbl3 *E. coli* at 30°C (Invitrogen, C737303). Templates for *in vitro* transcription were purified using the Plasmid Mega kit (Qiagen, 12181), linearized using XbaI restriction enzyme for full-length vRNA and RLuc replicon or AvrII (R0174S) for truncated vRNA, and purified by phenol–chloroform extraction and ethanol precipitation.

### RNA transcription and labeling

RNAs encoding 5′-3′ UTR, NanoLuc, and mRNA sequences were *in vitro* transcribed using the T7 Megascript kit (Invitrogen, AM1334) following the manufacturer’s protocol. Transcription products were purified using the GeneJET RNA Purification Kit (Thermo Fisher, K0732). The 3′ end was fluorescently labeled by potassium periodate oxidation and reaction with Cy3 hydrazide (Cytiva, PA13121) following published protocols [48]. 3′-Cy3 RNAs were capped with a Cap-1 m^7^G using the Vaccinia Capping System (NEB, M2080S) and 2′-*O*-methyltransferase (NEB, M0366), following the one-step protocol and purified using the GeneJET RNA Purification Kit. Capping efficiency was routinely >95% as assessed by protection from exonuclease-mediated degradation in a two step reaction with RNA 5′ polyphosphatase (Lucigen, RP8092H) and Terminator 5′-phosphate-dependent exonuclease (Lucigen, TER51020). The 5′-m^7^G was then fluorescently labeled by potassium periodate oxidation and reaction with Cy5 hydrazide (Cytiva, PA15121) to produce doubly labeled RNAs. RNAs were aliquoted, flash frozen on liquid N_2_, and stored at −80°C until use. RNA integrity and labeling was validated by denaturing polyacrylamide gel electrophoresis (PAGE) on 5% polyacrylamide-TBE gels containing 7.5 M urea after each reaction. Gels were imaged on a ChemiDoc Imaging System (Bio-Rad) by scanning for Cy3 and Cy5 fluorescence to visualize labeled RNAs and then stained with SYBR Green II (Invitrogen S7568) following manufacturer’s protocol to visualize total RNA.

Replication-deficient RLuc replicon and full-length vRNAs were *in vitro* transcribed using the T7 Megascript kit with the following modifications: linearized plasmid concentration in the reaction was 0.3 μg/μl, betaine was added to the reaction to 1 M final concentration, and reactions were incubated at 30°C for 4 h. Transcription products were purified by lithium chloride precipitation. Fluorescent labeling and capping were performed in the same order as above, with the following modifications. Periodate oxidation reactions contained 50 mM sodium periodate, 100 mM bis-Tris propane pH 7, and 5 mM EDTA and were carried out at room temperature for 1 h in the dark, followed by quenching with 66 mM ethylene glycol and 1 M betaine. Oxidized vRNAs were concentrated and buffer exchanged into a buffer containing 10 mM bis-Tris propane pH 7, 10 mM NaCl, and 0.1 mM EDTA using 30 kDa MWCO Amicon Ultra centrifugal filters (Amicon, UFC503024) spun at 1000 × *g* at least three times to remove oxidized ethylene glycol and excess salt. Reactions with hydrazide dyes contained 100 mM aniline to catalyze hydrazone formation [50], 100 mM bis-Tris propane pH 7, 0.5 units/μl SUPERase•In RNase inhibitor (Invitrogen, AM2696), and 5 mM EDTA. Fluorescently labeled vRNAs were concentrated and buffer exchanged at least three times as above to remove excess dye and aniline. Capping was performed with Faustovirus Capping Enzyme (NEB M2081L) and 2′-*O*-methyltransferase, following the one-step protocol with incubation at 30°C for 2 h. Capping reactions were quenched with 2 mM EDTA and capped vRNAs were purified by lithium chloride precipitation. Doubly labeled vRNAs were aliquoted, flash frozen on liquid N_2_, and stored at −80°C until use. RNA quality and labeling was validated after each reaction by denaturing agarose gel electrophoresis on 1% agarose gels cast with buffer containing 30 mM each trice and triethanolamine (1× TT buffer) and 0.4 M formaldehyde [51]. vRNA gel samples were prepared in buffer containing 50% (v/v) formamide, 0.4 M formaldehyde, 5 mM EDTA, and 1× TT and heated at 65°C for 5 min before loading. Gels were visualized as above.

### Native gel electrophoresis

WT DENV2 5′-3′ UTR (100 nM) was refolded and annealed to biotinylated RNA tethering oligonucleotide (1 μM) and CS-DAR or UAR DNA blocking oligonucleotides (50 nM–10 μM) by incubation at 65°C for 3 min followed by snap cooling on ice in a buffer containing 50 mM bis-Tris propane pH 7 and 100 mM potassium acetate. MgCl_2_ was added after cooling to a final concentration of 5 mM. Refolded RNAs were split into two samples for native PAGE and RNase H digestion. Loading buffer with a final concentration of 35 mM Tris–HCl pH 7.5, 65 mM HEPES-KOH pH 7.4, 5 mM MgCl_2_, and 10% (v/v) glycerol was added to native PAGE samples, which were kept on ice until loading. Samples were separated on 5% polyacrylamide gels cast with buffer containing 35 mM Tris–HCl pH 7.5, 65 mM HEPES-KOH pH 7.4, and 5 mM MgCl_2_ that were run in the same buffer at 4°C. RNase H samples were incubated with 0.5 units/μl RNase H (NEB, M0297S) at 30°C for 30 min in a buffer containing 20 mM HEPES-KOH, pH 7.4, 70 mM potassium acetate, 5 mM magnesium acetate, 0.25 mM spermidine, and 0.2 mg/ml creatine phosphokinase. Reactions were mixed with an equal volume of RNA Loading Dye, 2× (NEB, B0363S) and heated at 95°C for 2 min before loading onto 10% polyacrylamide-TBE gels containing 7.5 M urea. Both native and denaturing gels were visualized by staining with SYBR Green II (Invitrogen S7568) and imaged using a ChemiDoc Imaging System (Bio-Rad).

### Purification of human translation initiation factors

Recombinant eIF4A isoform 1 [52], eIF4B [48], eIF4E [53], and eIF4G (residues 165–1599) [48] were purified as described previously. Briefly, 6xHis-tagged eIF4A was expressed in BL21 (DE3) *E. coli* cells, lysed by sonication, and purified by Ni-NTA affinity chromatography. The 6xHis tag was removed by TEV protease cleavage and subtractive Ni-NTA purification before size-exclusion chromatography. 6xHis-tagged eIF4B and eIF4G were expressed in Sf9 insect cells and purified by the same method as eIF4A except that ion-exchange chromatography (heparin) was used instead of size-exclusion chromatography. eIF4E was expressed in Rosetta 2 (DE3) *E. coli* cells as a fusion protein with glutathione S-transferase (GST), lysed by sonication, and enriched using glutathione sepharose resin. The GST tag was removed by overnight digestion with PreScission protease, and eIF4E was eluted from the resin and purified by ion-exchange and size-exclusion chromatography.

### Single-molecule FRET measurements

Single-molecule fluorescence microscopy was performed at 19°C on a home-built, prism total internal reflection fluorescence (TIRF) microscope. Briefly, Cy3- (donor) and Cy5- (acceptor) labeled samples were excited using 532 and 640 nm solid-state lasers (Coherent OBIS™ LS Laser). Fluorescence emission was collected using a ×60 water-immersion 1.27 NA objective (Nikon) and was further cleaned up using a 532 nm longpass filter for single illumination experiments or a 488/532/640 nm notch dichroic mirror for dual illumination experiments. Emitted light was split onto two EMCCD cameras (Andor iXon Ultra 888) using a 640 nm dichroic mirror (Chroma Technology Corp, ZT640rdc). All data were collected at 10 frames per second with the EM gain set to 850.

RNAs were refolded as above except that the concentration of biotinylated RNA tethering oligonucleotide was 50 nM. Refolded RNAs were kept on ice until use. Biotinylated RNA complexes were diluted to 100–300 pM final concentration of the tethering oligonucleotide for short RNAs (<2 kb) in imaging buffer (20 mM HEPES-KOH, pH 7.4, 70 mM potassium acetate, 5 mM magnesium acetate, 0.25 mM spermidine, and 0.2 mg/ml creatine phosphokinase) and immobilized on the surface of neutravidin-coated quartz slides prepared as described previously [54] by incubation at room temperature for 5 min. vRNAs were immobilized at 1 nM final concentration of the tethering oligonucleotide and immobilized for 10 min at room temperature to allow for binding with slower diffusion of the larger biotinylated RNA complexes. Excess RNA was washed out with imaging buffer and the flow cell was exchanged into imaging buffer supplemented with 62.5 μg/ml casein and an oxygen scavenging system [54]: 2 mM TSY (Pacific Biosciences, 100-214-900), 2 mM protocatechuic acid, and 0.06 units/μl protocatechuate-3, 4-dioxygenase.

Single-molecule fluorescence imaging in zero-mode waveguide (ZMW) chips on a modified Pacific Biosciences RSII instrument was performed as described previously [48]. RNAs (2 nM) were immobilized on neutravidin-coated ZMW chips as above. Fluorescence movies were collected at 10 frames per second for 20 min at temperatures varying from 25°C to 37°C using a 532 nm excitation laser at 0.32 μW/μm^2^. For experiments with real-time fluidic delivery of factors, an equal volume of imaging buffer containing 1 mM ATP, 2 μM eIF4A, 440 nM eIF4B, 260 nM eIF4G, and 320 nM eIF4E was delivered to RNA immobilized on the chip in imaging buffer containing 1 mM ATP. As a result, the final protein concentrations during the experiment were 1 μM eIF4A, 220 nM eIF4B, 130 nM eIF4G, and 160 nM eIF4E.

### Single-molecule FRET data analysis

TIRF microscopy movies recording fluorescence intensities in the donor (Cy3) and acceptor (Cy5) channels were processed using SPARTAN version 3.7.0 [55] in MATLAB version R2023b to find colocalized molecules. Movies from ZMW-based experiments were processed using custom MATLAB scripts. For dual illumination experiments, molecules were initially selected by filtering all extracted fluorescence traces for those with Cy3 and Cy5 signals above background. Single molecules for analysis were manually selected for analysis based on observation of single step photobleaching events of both the donor and acceptor fluorophores during movie acquisition. Single molecule fluorescence traces were then binned into molecules with a single FRET-on state, molecules with transitions between FRET states, and molecules in a FRET-off state (i.e. no FRET signal observed). The number of traces in each bin were used to calculate the relative fraction of RNA molecules in each population.

For single illumination experiments, molecules with a FRET signal were initially selected by filtering all extracted fluorescence traces for those with a Pearson’s correlation coefficient between donor and acceptor fluorescence between −1.1 and −0.1. Single molecules for analysis were manually selected based on observation of single step photobleaching events of the donor fluorophore during movie acquisition. Background fluorescence intensities were corrected using SPARTAN and fluorophore crosstalk was corrected with a custom MATLAB script. FRET efficiency (*E*_FRET_) was defined as *E*_FRET_ = *I*_A_ / (*I*_D_ + *I*_A_) where *I*_D_ and *I*_A_ are the intensity of the donor and acceptor channels, respectively. With doubly labeled RNAs, we observed low intensity excitation of Cy5 in single illumination experiments that gave an apparent *E*_FRET_ value of ∼0.1 despite not being anticorrelated with Cy3 intensity. For this reason, we excluded states with *E*_FRET_ ≤ 0.1 from analysis. FRET states were assigned automatically using vbFRET version June10 [56].

To estimate a combined E_FRET_ distribution of all observed molecules, FRET traces from single illumination data were separated into (i) molecules with a single FRET state observed from the beginning of the movie until photobleaching of fluorescent dyes or (ii) molecules with multiple FRET states observed by fluctuation between FRET-off and FRET-on states and/or transitions between two or more FRET-on states. Observation of *E*_FRET_ values in group 1 was assumed to be limited by photobleaching of Cy5; so *E*_FRET_ values were extracted from only the first 50 frames of each trace to ensure that each molecule would be equally represented in the *E*_FRET_ distribution and that the distribution would not be skewed towards molecules with longer Cy5 lifetimes. For molecules in group 2, *E*_FRET_ values observed across all FRET events and states were extracted. *E*_FRET_ values for the FRET-off state were estimated by extracting *E*_FRET_ values for molecules that had a single Cy3 dye but exhibited no FRET events. The frequencies of *E*_FRET_ values obtained for group 1 traces, group 2 traces, and the FRET-off state were normalized by the fraction of molecules belonging to each of these populations as observed in the dual illumination experiments. *E*_FRET_ values were then combined and binned (50 bins, −0.2 < *E*_FRET_ < 1.2) and fit to Gaussian functions.

### Statistical analysis

Errors reported for rate constants represent the 95% confidence interval of the single or double exponential fit obtained using the cftool package in MATLAB. Errors reported for median lifetimes and relative population fractions are the 95% confidence interval obtained by bootstrapping analysis using the bootci function in MATLAB. *E*_FRET_ values of conformational states are reported as the mean and standard deviation of the Gaussian distribution fit. Statistical differences between mean *E*_FRET_ values were calculated using a two sample *Z*-test for means.

## Results

### Orthoflavivirus vRNA conformation is heterogeneous

**Figure 2.**
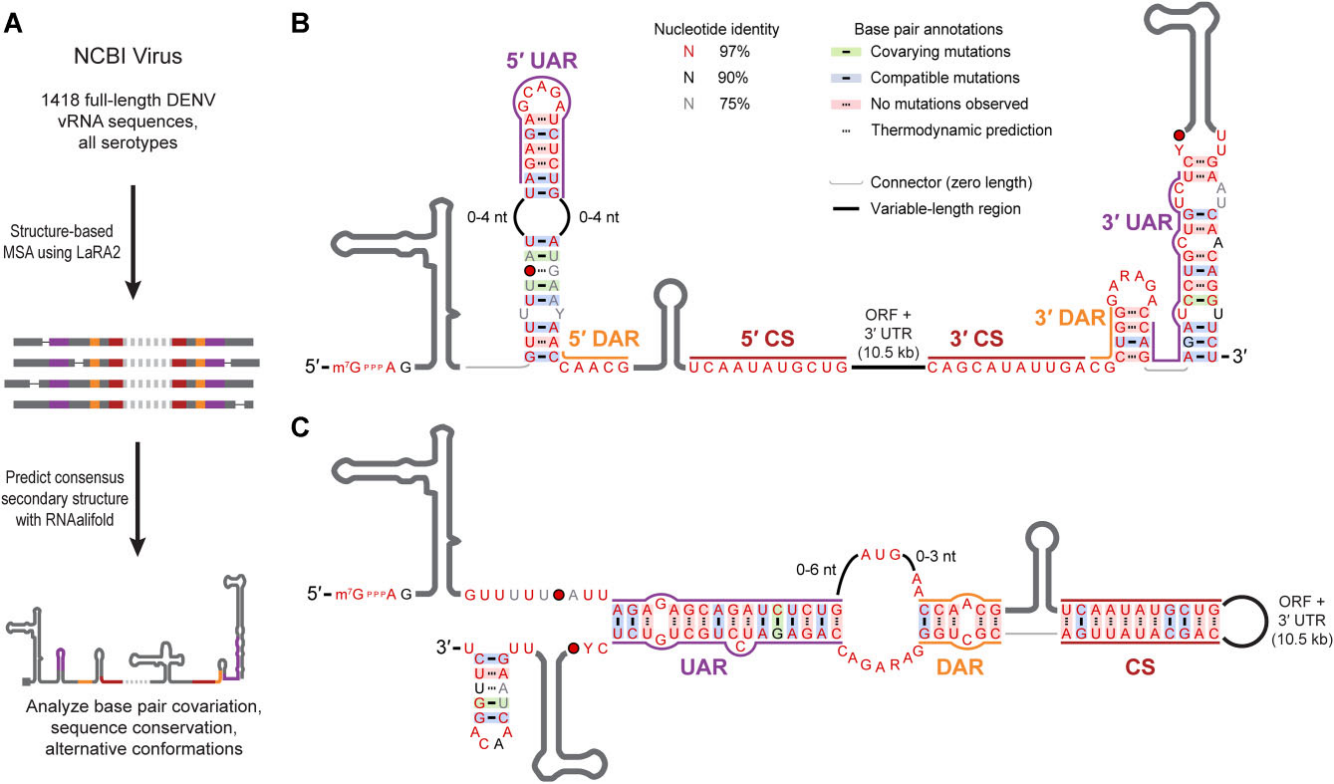
The extended and cyclized conformations of DENV vRNA are evolutionarily conserved. (**A**) Overview of pan-serotype bioinformatic analysis of DENV sequence and structure conservation. (**B**) Sequence-structure conservation diagram of the proposed extended conformation of DENV vRNA. Sequence conservation and base pair covariation for 1418 sequences were analyzed and diagram prepared with R2R [46]. Base pairs supported by phylogenetic analysis are shown with a solid line, and base pairs that are only supported by secondary structure prediction are indicated with a dashed line. Evolutionarily supported base pairs were annotated as covarying and highlighted in green if both nucleotides changed identities in one or more strains (e.g. G-C to A-U), and were annotated as compatible and highlighted in blue if just one residue changed (e.g. G-C to G-U). Predicted base pairs between universally conserved nucleotides are highlighted with a red box. The font color of individual residues indicates primary sequence conservation. R and Y stand for positions that must be purines or pyrimidines, respectively. A filled red circle indicates a residue that is absent (gap in the MSA) in ≤3% of DENV sequences analyzed. The first start codon does not appear to be well conserved because its position within the lower portion of SLB varies between serotypes. (**C**) The same MSA shown in panel (B) was analyzed in the context of the proposed cyclized conformation of vRNA.

Since RNA viruses rapidly evolve and pathogens of concern are regularly surveilled by sequencing, we explored whether there is phylogenetic support for both proposed orthoflaviviral vRNA conformations in circulating viral strains. We analyzed the sequence and structure conservation of DENV because of the large number of genetically diverse sequences accumulated in databases from outbreaks over several decades. All unique, full-length DENV vRNA sequences available on NCBI Virus were downloaded, trimmed to the 5′ and 3′ terminal regions, and a multiple sequence alignment (MSA) was performed with LaRA2, which utilizes both sequence and secondary structure information (Fig. 2A) [44]. The final MSA contained 1418 vRNA sequences covering all four DENV serotypes (Fig. 2B and C, and Supplementary Fig. S1) [46]. Although the UAR, DAR, and CS are nearly universally conserved, there is evolutionary support for both alternative secondary structures of the terminal regions, with covarying base pairs observed in local (SLB and 3′-SL) and long-range (5′-3′ UAR) interactions.

**Figure 3.**
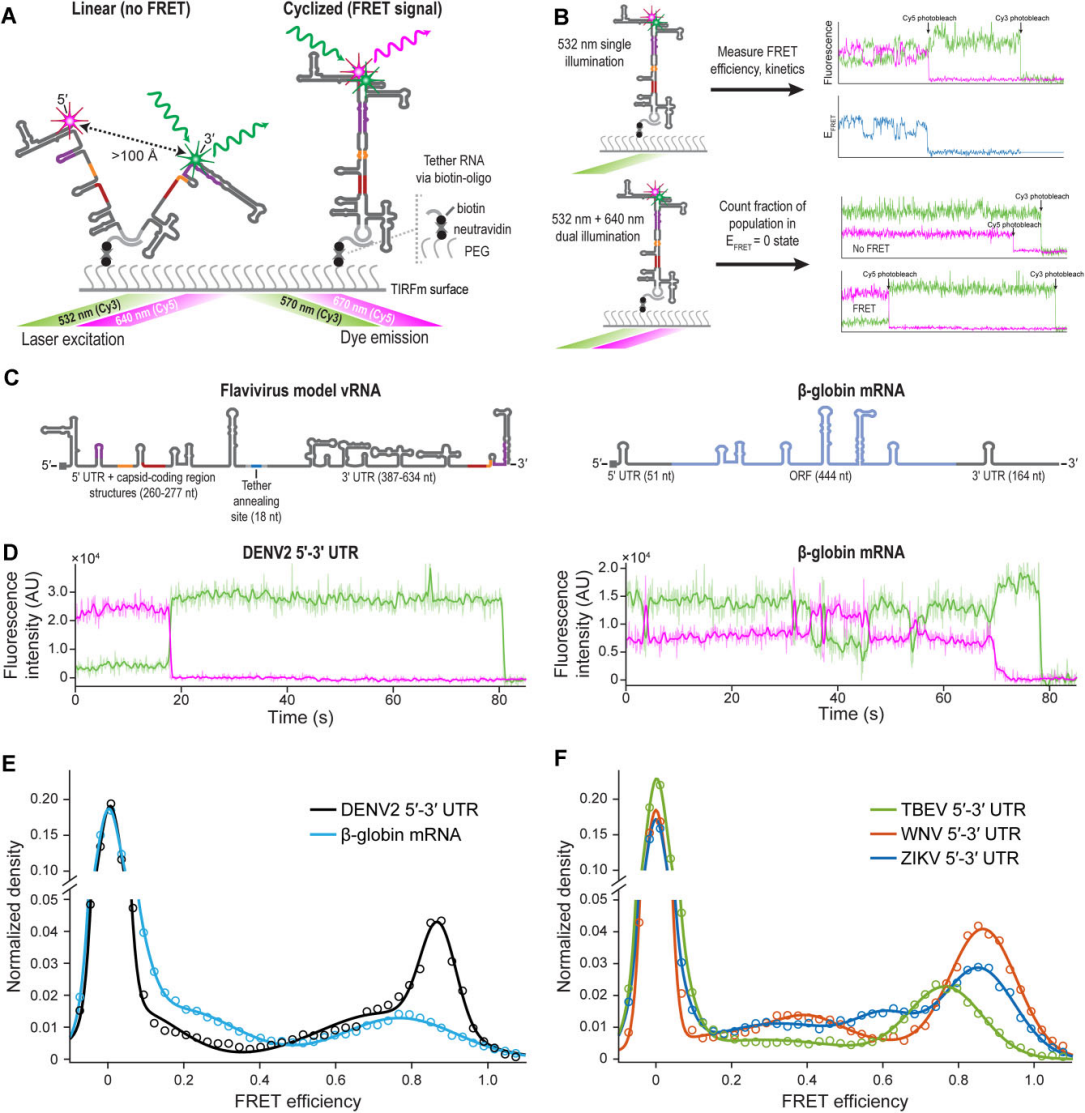
Orthoflaviviral RNA and mRNA conformations are heterogeneous. (**A**) Schematic of the 5′-3′ smFRET experiment using two-color TIRF microscopy. (**B**) Overview of approach to probe RNA conformational ensembles using smFRET. Single illumination experiments identify different conformational states distinguishable by 5′-3′ end distance and measure the kinetics of exchange between these states. Dual illumination experiments count the number of doubly labeled molecules that either display 5′-3′ FRET or remain in *E*_FRET_ = 0 states for the duration of the observation window. (**C**) Cartoon schematic of minigenome and mRNA constructs analyzed. Positions of conserved complementary sequences are highlighted with the UAR in purple, DAR in yellow, and CS in maroon. The ORF of β-globin mRNA is colored in light blue. (**D**) Representative 5′-3′ smFRET traces collected with 100 ms exposure time for the DENV2 5′-3′ UTR and β-globin mRNA. The single-step drops in magenta and green fluorescence intensity for DENV2 5′-3′ UTR are due to photobleaching of Cy5 and Cy3, respectively. The solid lines are fluorescence counts smoothed with a sliding window over *n* = 5 frames. Raw data are displayed in light coloring. (**E**) FRET efficiency distribution of DENV2 5′-3′ UTR and β-globin mRNA. *n* = 104 and *n* = 233 single illumination and *n* = 309 and *n* = 306 dual illumination traces were analyzed for each RNA, respectively. The solid lines are the Gaussian fit to the observed data. (**F**) FRET efficiency distribution for representative orthoflaviviral 5′-3′ UTR sequences, prepared as in panel (E). Number of single illumination and dual illumination traces analyzed for each construct, respectively, were *n* = 131 and *n* = 161 for TBEV 5′-3′ UTR, *n* = 174 and *n* = 163 for WNV 5′-3′ UTR, and *n* = 303 and *n* = 397 for ZIKV 5′-3′ UTR.

We hypothesized that the proposed alternative conformations of vRNA (Fig. 1) could be distinguished by the distance between the 5′ and 3′ ends. To measure the end-to-end distance using FRET, we labeled *in vitro* transcribed RNAs with Cy5 and Cy3 on the m^7^G cap and 3′ end, respectively, using periodate oxidation and hydrazide dyes (“Materials and methods” section, Supplementary Fig. S2). We then utilized smFRET imaging to investigate the conformational heterogeneity of representative vRNA and mRNA samples (Fig. 3A and B). To study the conformation of orthoflavivirus vRNA terminal regions we examined minigenome constructs (Fig. 3C and Supplementary Table S2), which have previously been used to investigate viral translation and genome replication *in vitro* [7, 26]. The minigenome constructs contained the 5′ UTR and capsid coding region structures implicated in regulation of the 5′-3′ long-range interactions [33, 57, 58], separated from the full 3′ UTR by a short unstructured linker sequence composed of an annealing site (18 nt) for a biotinylated oligonucleotide flanked by single-stranded spacers (10 nt each). Dual-labeled RNAs complexed with the tethering oligonucleotide (Supplementary Table S1) were immobilized on neutravidin-coated quartz slides and imaged with a prism-based TIRF microscope (Fig. 3A and Supplementary Fig. S3A). Upon illumination with a 532 nm laser, single-molecule fluorescence traces of individual RNA molecules displayed 5′-3′ FRET (“FRET-on” states, Fig. 3D).

The orthoflavivirus 5′-3′ UTR constructs adopted multiple conformations. In the case of the DENV2 5′-3′ UTR, two states fit to well-resolved Gaussian distributions with *E*_FRET_ = 0.00 ± 0.03 and *E*_FRET_ = 0.87 ± 0.05 (Supplementary Table S3). Less frequent intermediate *E*_FRET_ values could be approximated by a much broader distribution (σ = 0.17) centered at *E*_FRET_ = 0.73 that may encompass multiple minor FRET-on states. Based on the observed FRET efficiencies, the end-to-end interdye distances ranged from a further 5–7 nm distance (“low FRET” states) to relatively close 5′-3′ proximity of <4 nm (“high FRET” state, Fig. 3E and F). For all four representative viral sequences, the high FRET state was the predominant FRET-on conformation (Fig. 3E and F).

Over our observation period, transitions between FRET states were observed only in a minority of traces (∼10% or less) and measured FRET lifetimes for DENV2 were limited by photobleaching of Cy5 (Supplementary Fig. S3B and C). In contrast, a 659 nt model human mRNA, β-globin, was conformationally dynamic (Fig. 3D). Transitions between three states centered at *E*_FRET_ = 0.00 ± 0.03, *E*_FRET_ = 0.2 ± 0.15, and *E*_FRET_ = 0.8 ± 0.15 states were observed. Transition rates between any two states were biphasic with *k*_obs, slow_ ≈ 0.2–0.9 s^−1^ and *k*_obs, fast_ ≈ 2–6 s^−1^ (Supplementary Fig. S3E), suggesting that the observed *E*_FRET_ peaks may represent more than one RNA conformation or that there may be multiple pathways of interconversion between states.

We hypothesized that the global conformation of RNAs would be heterogeneous, where some molecules have 5′ and 3′ ends in close proximity (i.e. cyclized vRNA) and others have their ends very distal, beyond the range of Cy3–Cy5 FRET. To test this model, we directly excited tethered RNAs with both 532 and 640 nm lasers (Fig. 3B). We quantified how many RNAs contained both dyes and of that population, how many yielded FRET. We observed that 45–60% of β-globin mRNA or orthoflaviviral RNAs lacked FRET between the Cy3- and Cy5-labeled RNA ends (Supplementary Fig. S3D), indicating that these molecules had end-to-end distances >8 nm (“FRET-off” state(s)). Native gel electrophoresis analysis of the DENV2 5′-3′ UTR also revealed two species with different electrophoretic mobility (Supplementary Fig. S4A, “RNA only” lane). Both species appeared to be monomeric based on comparison with ssRNA size standards, suggesting that the differing mobility is due to alternative intramolecular interactions, consistent with our observation of both high FRET and FRET-off states of individual RNA molecules. The relative intensity of each band was also comparable to the observed occupancy of high FRET and FRET-off states in smFRET experiments (Supplementary Fig. S4D), supporting our assignment of the faster migrating species as a cyclized conformation and the slower migrating species as an extended conformation.

Lowering the monovalent ion concentration reduced the frequency of the DENV2 5′-3′ UTR high FRET state (Supplementary Fig. S5A). However, increasing the concentration of K^+^ >100 mM did not lead to further increases in the fraction of cyclized RNA beyond 44 ± 6%. These results suggest that the FRET-off and FRET-on states represent alternative secondary structures, as we observed a constant ratio of the frequency of the states at K^+^ concentrations >50 mM. Stable folding of FRET-on states also required the presence of Mg^2+^. In the absence of Mg^2+^, we observed rapid fluctuations between FRET states in 29 ± 4% of immobilized doubly labeled RNAs and a broad *E*_FRET_ distribution (Supplementary Fig. S5B–D). FRET-on states were stabilized by the addition of only 0.5mM Mg^2+^ with only a slight dependence of the fraction of RNA in FRET-on states as the concentration of Mg^2+^ was raised further. Only 32 ± 5% of DENV2 5′-3′ UTR adopted FRET-on states after refolding and imaging in the presence of 0.5 mM Mg^2+^, compared with 54 ± 5% in 10 mM Mg^2+^ (Supplementary Fig. S5B). The position of the high FRET peak shifted from *E*_FRET_ = 0.80 ± 0.09 to 0.7 ± 0.12 with 0.5 mM Mg^2+^ (Supplementary Fig. S5D), indicating a larger end-to-end distance and a less compact cyclized conformation. Together, these data suggest that folding of a stable cyclized conformation with close end-to-end distance requires Mg^2+^, which is a key facilitator of RNA tertiary contacts [59].

### CS interaction is important for short end-to-end distance

**Figure 4.**
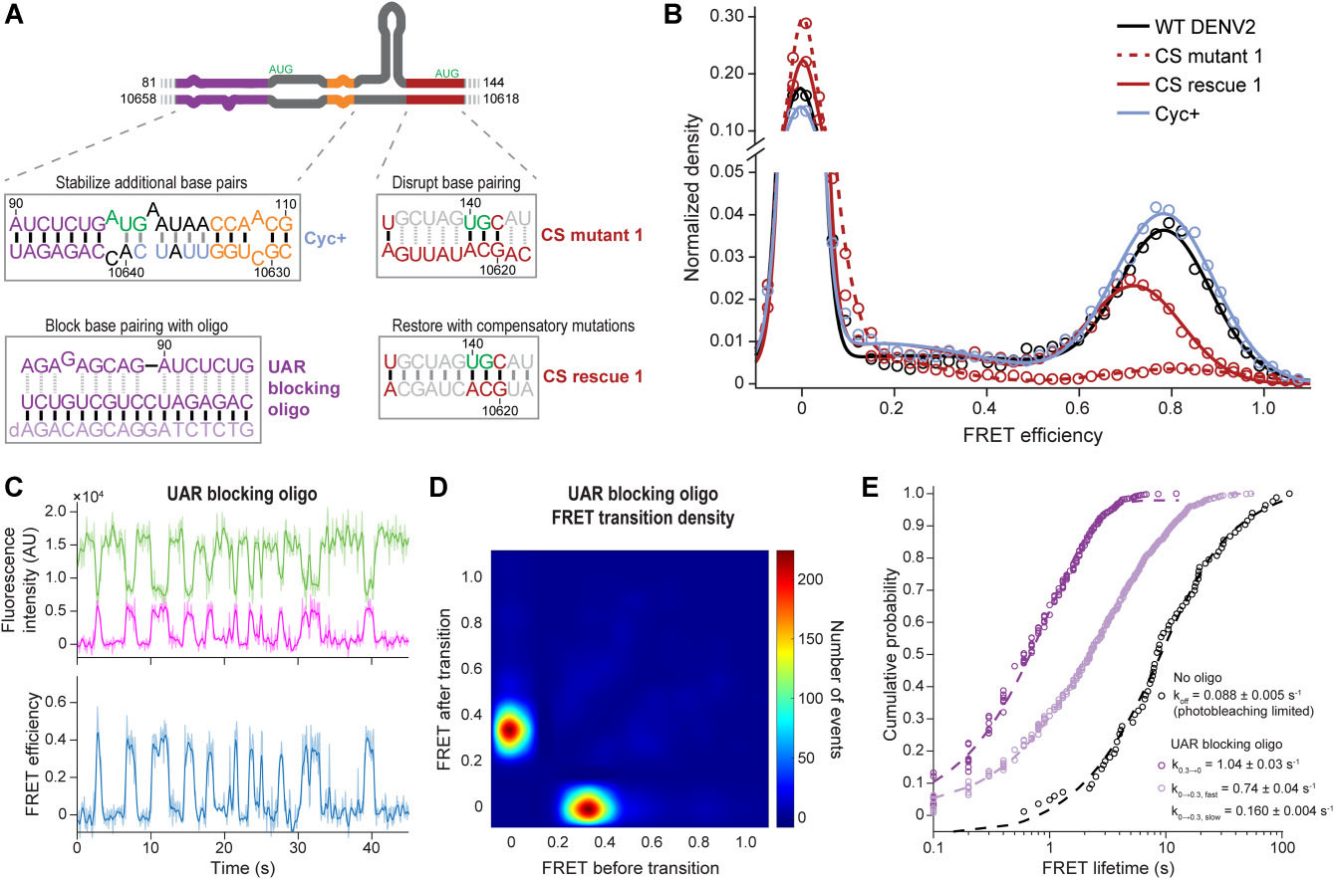
Stable 5′-3′ FRET states depend on long-range interactions. (**A**) Overview of perturbations used to probe the functions of conserved complementary sequences. Examples shown (clockwise from top left) are stabilization of the cyclized state with additional base pairs, disruption and reconstitution of 5′-3′ CS base pairing, and inhibition of the 5′-3′ UAR interaction with a DNA oligonucleotide. Binding of the blocking oligonucleotides to the predicted positions in the 3′ UTR was validated by digesting the RNA–DNA duplex with RNase H and observing the resulting RNA fragments by denaturing gel electrophoresis (Supplementary Fig. S4B and C). (**B**) The high FRET state is stabilized by long-range base pairing. FRET efficiency distributions of representative DENV2 5′-3′ UTR mutants are plotted with open circles. The solid lines are the Gaussian fit to the observed data. Number of single illumination and dual illumination traces analyzed for each construct, respectively, were *n* = 114 and *n* = 309 for WT DENV2 5′-3′ UTR, *n* = 99 and *n* = 359 for CS mutant 1 5′-3′ UTR, *n* = 104 and *n* = 314 for CS rescue 1 5′-3′ UTR, and *n* = 196 and *n* = 263 for Cyc + 5′-3′ UTR. (**C**) Representative 5′-3′ smFRET trace for the DENV2 5′-3′ UTR with the 3′ UAR blocking oligonucleotide annealed. Top, Cy3 and Cy5 fluorescence intensity; bottom, FRET efficiency. The solid lines are fluorescence counts smoothed with a sliding window over *n* = 3 frames. Raw data are displayed in light coloring. To highlight rapid FRET state transitions, the trace is zoomed in on the region before Cy5 photobleaching and Cy3 photobleaching is not shown. (**D**) Transition density plot analysis of 5′-3′ smFRET in the presence of the 3′ UAR blocking oligonucleotide. 2662 transitions from 187 individual smFRET traces were observed and plotted. (**E**) Kinetics of DENV2 5′-3′ UTR FRET transitions. Observed photobleaching-limited lifetimes of the FRET state in the absence of blocking oligonucleotide s are plotted as a cumulative probability function in black circles. Observed lifetimes of the *E*_FRET_= 0 and *E*_FRET_= 0.3 states in the presence of the 3′ UAR blocking oligonucleotide are plotted as cumulative probability functions in light and dark purple circles, respectively. The data were fit to single or double exponential equations to extract rate constants. The error reported represents the 95% confidence interval (C.I.) of the fit. For the UAR blocking oligonucleotide *k*_0→0.3_ fit, the amplitudes of the fast and slow phases were 40% and 60%, respectively.

We hypothesized that the stable, high FRET conformation of the viral RNAs results from specific long-range base pairing, rather than general intrinsic 5′-3′ proximity caused by compaction of the RNA by secondary structure. To test this model, we first disrupted the complementarity of the CS sequences in the well-characterized DENV2 16681 strain (Fig. 4A and Supplementary Fig. S6A). Mutation of either 7 or 6 nucleotides in the 5′ or 3′ UTR (CS mutant 1 and CS mutant 2, respectively) to noncomplementary bases reduced the fraction of RNA in a stable FRET-on state from 36 ± 6% to 8 ± 3% or 11 ± 4% (Fig. 4B and Supplementary Fig. S6B). Restoring the complementarity of the CS by introducing compensatory mutations into the other UTR partially rescued this decrease in the FRET-on population (Fig. 4B). In parallel, a DNA oligonucleotide annealed to the 3′ CS region in the WT construct reduced the prevalence of the FRET-on state similarly to the CS mutants (Supplementary Fig. S6B). Inhibition of the long-range interaction also prevented formation of the faster migrating species observed by native gel electrophoresis (Supplementary Fig. S4A).

Since disruption of base pairing reduced the FRET-on state, we predicted that enhancing long-range base pairing would increase the frequency of the FRET-on state. We introduced six additional long-range base pairs in an unpaired region between the 5′-3′ DAR and 5′-3′ UAR by changing the small hairpin (sHP) loop sequence in the 3′ UTR from GAAAGA to UUAUCA (Fig. 4A). Indeed, these substitutions increased the fraction of RNAs in FRET-on states from 41 ± 6% to 51 ± 6% (Supplementary Fig. S6B). Collectively, short end-to-end distances (i.e. 5′-3′ FRET) thus are stabilized by the 5′-3′ CS interaction in the DENV2 construct, which very likely represents the cyclized vRNA state. vRNAs in a FRET-off state likely represent one or more extended conformations.

### UAR base pairing is required for stable 5′-3′ end proximity

While the 5′-3′ CS interaction is only incompatible with a pseudoknot between the 3′-CS and a loop in the 3′ dumbbell (3′-DB) structure (Supplementary Fig. S1C), formation of the 5′-3′ UAR interaction would require more extensive local secondary structure rearrangements. Long-range base pairing must replace SLB in the 5′ UTR as well as sHP and the base of 3′-SL in the 3′ UTR (Fig. 1B). We designed two variants of the DENV2 5′-3′ UTR by incorporating 5′-UAR mutations that were previously shown to abrogate viral replication [14]. These variants are predicted to disrupt the 5′-3′ UAR and DAR interactions, reducing the average base pairing probability of these interactions to ≤1% (Supplementary Fig. S6E). Disruption of the 5′-3′ UAR interaction by either these sequence changes or annealing a DNA oligonucleotide to the 3′-UAR slightly decreased the fraction of DENV2 5′-3′ UTR RNAs in FRET-on states from 41 ± 6% to 36 ± 5%, 35 ± 4%, or 26 ± 3% (Supplementary Fig. S6F). Sequence mutations slightly shifted the position of the high FRET state (Supplementary Fig. S6G) from *E*_FRET_ = 0.8 ± 0.10 in WT to *E*_FRET_ = 0.7 ± 0.13 in UAR mutant 1 (*P* = 5.0 × 10^–6^), while the shift in UAR mutant 2 (*E*_FRET_ = 0.77 ± 0.09) was statistically insignificant relative to WT. Overall, UAR disruption by sequence substitutions did not inhibit 5′-3′ FRET as severely as 5′-3′ CS disruption.

In contrast to the sequence variants, which disrupt just 3–4 long-range UAR base pairs (Supplementary Fig. S6A), complete inhibition of the 5′-3′ UAR interaction by the DNA blocking oligonucleotide altered DENV2 5′-3′ UTR conformational dynamics (Fig. 4C). The majority of RNAs in a FRET-on state were observed to fluctuate between two states with E_FRET_ = 0.00 ± 0.03 and 0.29 ± 0.09 (Fig. 4D). The lower *E*_FRET_ value we observed for the FRET-on state in the presence of the blocking oligonucleotide relative to UAR mutant 1 and UAR mutant 2 may be due to the intermolecular RNA–DNA duplex formed. This could lengthen the distance between the 5′-3′ CS and/or DAR stem(s) and the 3′ end, increasing the end-to-end distance compared with constructs in which the intramolecular 3′-SL keeps the 3′-Cy3 in closer proximity to other complementary sequences and thus the 5′-Cy5. We observed a similar result by native gel electrophoresis, with reduced intensity of both bands and smearing between them, suggestive of increased conformational heterogeneity (Supplementary Fig. S4A).

DENV2 5′-3′ UTR bound by the 3′-UAR blocking oligonucleotide fluctuated between the two states with rate constants *k*_0→0.3, fast_ = 0.74 ± 0.04 s^-1^, *k*_0→0.3, slow_ = 0.160 ± 0.004 s^-1^, and *k*_0.3→0.1_ = 1.04 ± 0.03 s^-1^ (Fig. 4E). Since the median lifetime of the FRET-on state is 2.4 ± 0.3 min at 37°C (Supplementary Fig. S3C) and likely even longer at room temperature, loss of the 5′-3′ UAR interaction increases the rate of transition from the FRET-on to FRET-off states ≥200-fold. These results show that although the 5′-3′ CS and DAR interactions alone can establish a cyclized state, the 5′-3′ UAR interaction is required for the high kinetic stability of this conformation. Importantly, a similar fraction of RNA exclusively occupied the FRET-off state(s) and displayed no FRET transitions in the presence or absence of sequence mutations or the blocking oligonucleotide, suggesting that the stability of the extended state(s) was unaffected by the inhibition of the 3′ UAR.

### Closely related DENV serotypes have diverse conformational ensembles

DENV has four distinct serotypes (DENV1–4) with highly similar RNA secondary structures in the terminal regions, including near-universal conservation of the CS, DAR, and UAR (Fig. 2B and C). To test whether vRNA conformations are shared among these closely related viral sequences, we measured 5′-3′ FRET in minigenome constructs based on representative strains from each serotype. As with DENV2, the major FRET-on conformation of DENV3 and DENV4 5′-3′ UTRs was a high FRET state (Supplementary Fig. S7A). However, with DENV1, we observed a shift from the predominant high FRET state conserved between all other orthoflaviviral sequences tested to two low FRET conformations at *E*_FRET_ = 0.2 ± 0.12 and 0.4 ± 0.11 (Supplementary Fig. S7A and B). In addition, the DENV1 5′-3′ UTR was conformationally dynamic, with more than half of all single-molecule traces yielding 5′-3′ FRET having one or more FRET transitions (Supplementary Fig. S7C). The DENV1 5′-3′ UTR predominantly dwelled in the low FRET states, with only brief excursions to conformations with closer end-to-end distances. The DENV3 and DENV4 5′-3′ FRET ensembles were intermediate, with a higher prevalence of conformational dynamics (21 ± 5% and 12 ± 3% of all single-molecule traces) compared to DENV2 (6 ± 3%, Supplementary Fig. S7D), but a greater frequency of the high FRET state relative to DENV1 (Supplementary Fig. S7B).

We observed no differences in the consensus CS, DAR, and UAR sequences between DENV1 and DENV2 strains. However, two substitutions each in SLB and 3′-SL are conserved in all DENV1 strains and are predicted to stabilize SLB and 3′-SL relative to DENV2 by 3 kcal/mol and 1.2 kcal/mol, respectively (Supplementary Fig. S7E). We hypothesized that collectively these mutations might destabilize the long-range 5′-3′ UAR interaction in DENV1 relative to these competing local structures, favoring a conformation in which the 5′-3′ CS and possibly DAR interactions have formed but the UAR has maintained its local secondary structures. However, introducing the predicted SLB- and 3′-SL-strengthening or -weakening mutations in DENV2 and DENV1, respectively, had only minor effects on their conformational ensembles (Supplementary Fig. S7F). Our results suggest that inter-serotype differences in the conformational ensemble are not explained by predicted differences in thermodynamic stability of the 5′ and 3′ UAR secondary structure elements that must unfold for long-range base pairing to occur. The conformational differences between DENV1 and DENV2 may require the cumulative effect of these substitutions with others more distal to the conserved complementary sequences.

### Full-length DENV2 vRNA is cyclized by long-range interactions

**Figure 5.**
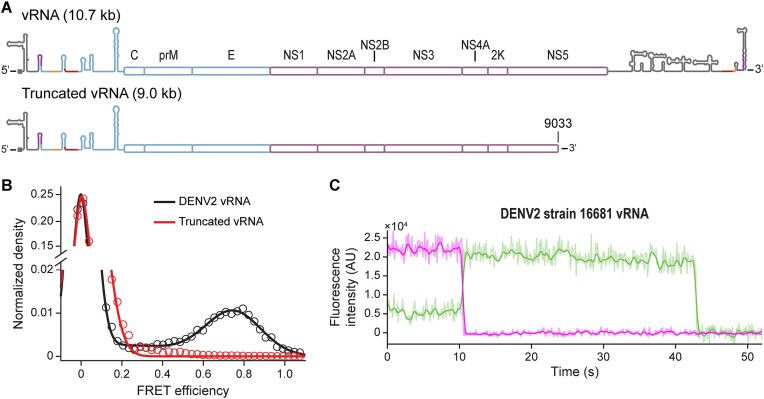
Cyclization of full-length DENV2 vRNA. (**A**) Full-length and truncated DENV2 strain 16681 vRNA constructs analyzed by 5′-3′ smFRET. ORFs are shown as boxes proportional to the length of the sequence. The vRNA polyprotein ORF is subdivided into the protein products produced by proteolytic cleavage during infection. Terminal regions are shown as cartoon structures, not to scale with the ORF. (**B**) FRET efficiency distribution of doubly-labeled constructs shown in panel (A). *n* = 90 single illumination and *n* = 423 dual illumination traces were analyzed for full-length vRNA and *n* = 23 single illumination and *n* = 350 dual illumination traces were analyzed for truncated vRNA. (**C**) Representative 5′-3′ smFRET trace for the full-length DENV2 vRNA, showing a single stable high FRET state that persists until photobleaching of Cy5. The solid lines are fluorescence counts smoothed with a sliding window over *n* = 5 frames. Raw data are displayed in light coloring.

While minigenome constructs have been useful tools for biochemical studies of vRNA, they remove ∼95% of the native sequence context and potentially bias the conformational ensemble towards the cyclized state because of shorter biopolymer length. The absent vRNA ORF contains many regions with functional RNA structures [35, 38, 40], including other potential long-range base pairing interactions (>1000 nt apart in primary sequence) [36, 41]. To test the effect of these other vRNA regions on 5′-3′ FRET, we prepared dual-labeled DENV2 RNAs with increasing distance between the terminal regions, up to the 10.7 kb full-length vRNA (Fig. 5A and Supplementary Fig. S8A).

Preparation of the DENV2 RLuc replicon and vRNA required modified transcription, purification, capping, and labeling protocols (“Materials and methods” section and Supplementary Fig. S8B). We found that the long vRNA sequences were prone to aggregation, forming a hydrogel during centrifugation and long incubations that could be solubilized by heating to 37°C in the presence of 1 M betaine. Acidic pH reproducibly caused this phase transition, leading us to carry out labeling chemistry in pH 7 buffer. Periodate oxidation was less efficient and hydrazide dye labeling was significantly slower than with shorter constructs, yielding labeling efficiencies <10% with standard protocols. Increasing periodate concentration to 50 mM during oxidation and using aniline to catalyze hydrazone formation [50] significantly improved labeling efficiency, allowing us to routinely achieve > 50% labeling efficiency on the cap and 3′ end. 5′-m^7^G capping with the vaccinia capping enzyme was also inefficient, while the faustovirus capping enzyme provided more robust results. Finally, we observed that the long vRNAs were prone to degradation. Quenching Mg^2+^-containing reactions (e.g. *in vitro* transcription and enzymatic capping) with excess EDTA and inclusion of RNase inhibitors during long incubations prevented this degradation (Supplementary Fig. S8B).

All three of the longer constructs tested had FRET-on states similar to the DENV2 5′-3′ UTR, with a high FRET state at *E*_FRET_ ≈ 0.75 ± 0.14 being the predominant FRET-on conformation (Fig. 5B and C, and Supplementary Fig. S9B). We observed that FRET-on states were less common in long vRNAs (16 ± 4% in full-length vRNA; Supplementary Fig. S9C), suggesting that either vRNA can fold into additional FRET-off states with native sequence context or that the cyclized conformation is less stable relative to the extended state when compared to the minigenome constructs. It is also possible that long vRNAs are prone to misfolding *in vitro* in the absence of host and viral RNA chaperones, especially since we experienced persistent aggregation during their preparation. We obtained similar results when we immobilized vRNA with an oligonucleotide annealed to an unstructured region in the Env ORF (Supplementary Fig. S9D), indicating that global vRNA conformation is unaffected by oligonucleotide binding. As a control, we synthesized a truncated 9.0 kb vRNA lacking the 3′ UTR (Fig. 5A) and observed a different distribution of FRET-on states (Fig. 5B) with low 5′-3′ FRET frequency in the population (4 ± 2% of all single-molecule traces; Supplementary Fig. S9C). Additionally, the few truncated vRNA traces we found that yield FRET underwent dynamic transitions (Supplementary Fig. S9E), supporting the importance of conserved complementary sequences for stable 5′-3′ end proximity in full-length DENV2 vRNA.

### Translation initiation factors rapidly remodel cyclized vRNA

**Figure 6.**
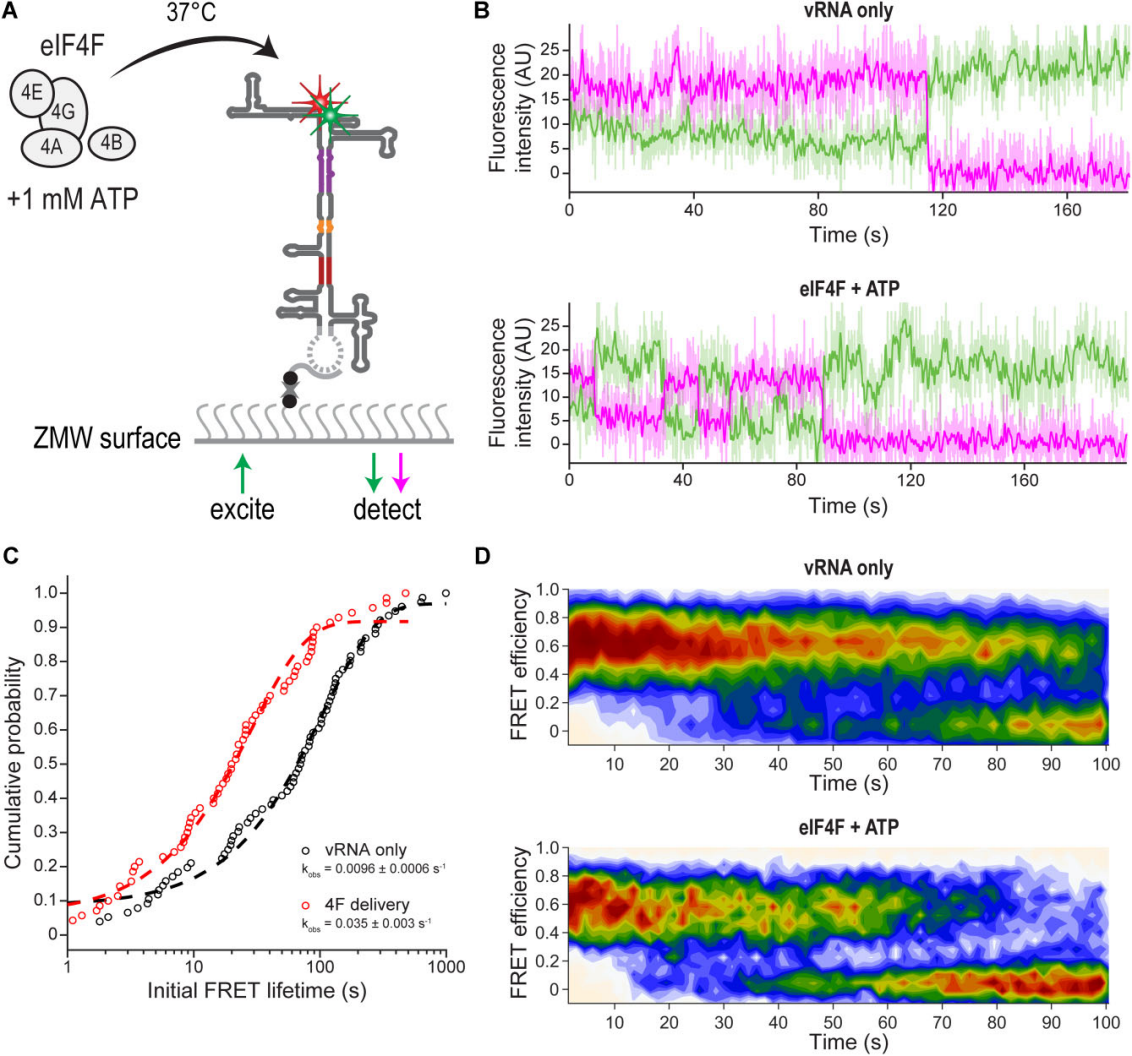
Conformational remodeling of DENV2 vRNA by eIF4 proteins. (**A**) Schematic of eIF4 delivery experiments on the custom RS instrument. The final concentrations of protein factors after delivery were 1 μM eIF4A, 220 nM eIF4B, 130 nM eIF4G, and 160 nM eIF4E. (**B**) Representative 5′-3′ smFRET traces for the full-length DENV2 vRNA in the presence and absence of eIF4 proteins. Buffer or factors were delivered at*t* = 0 s. The solid lines are fluorescence counts smoothed with a sliding window over *n* = 5 frames. Raw data are displayed in light coloring. (**C**) Initial FRET lifetime observed after delivery of either buffer or eIF4 proteins in the presence of 1 mM ATP. *n* = 108 traces were analyzed for the vRNA only condition, and *n* = 78 traces were analyzed for eIF4F + ATP delivery. The data were fit to single exponential equations to extract rate constants. The error reported represents the 95% CI of the fit. (**D**) Density maps of normalized doubly-labeled vRNA FRET efficiency after buffer or factor delivery for traces quantified in panel (C).

The vRNA interacts with a changing cast of RNA-binding proteins (RBPs) to function in protein synthesis, genome replication, and packaging. In particular, host RBPs are essential for translation initiation by selecting mRNAs to be translated and preparing them for loading onto the ribosome. These comprise eIF4A, a DEAD-box RNA helicase; eIF4B, a cofactor that binds RNA and stimulates eIF4A activity; eIF4E, the m^7^G cap-binding protein; and eIF4G, a scaffolding protein that binds RNA, eIF4A, and eIF4E [60]. To understand whether these RBPs can preferentially stabilize certain conformational states of orthoflaviviral vRNA, we immobilized minigenome and full-length DENV2 vRNAs on the surface of ZMW chips to measure 5′-3′ FRET after real-time delivery of purified human eIF4 proteins (Fig. 6A).

We observed a rapid loss of 5′-3′ FRET in cyclized DENV2 5′-3′ UTR after delivery of all four eIF4 proteins at physiological concentrations in the presence of 1 mM ATP (Supplementary Fig. S10A and B), with a median lifetime of 8.0 s (95% CI: 7.5–8.8 s). This was significantly shorter than the photobleaching-limited lifetime of DENV2 5′-3′ UTR when imaged in the absence of proteins (Supplementary Figs S3C and S10B). eIF4 proteins also induced a change in DENV2 vRNA conformation that was faster than the photobleaching rate (Fig. 6B–D). With full-length vRNA we observed additional transitions between low FRET or FRET-off states and high FRET states following the initial disappearance of 5′-3′ FRET that did not occur for protein-free vRNA (Fig. 6B and D). For both vRNA constructs, we did not see the appearance of FRET in RNA molecules that were in an extended conformation(s) prior to the delivery of factors. These results suggest that the activity of eIF4 proteins promotes the reversible remodeling of cyclized vRNA to an extended conformation, and that this remodeling activity is specific for the cyclized conformation.

Because eIF4A is reported to have RNA unwinding activity, we tested whether eIF4A alone could mediate DENV2 5′-3′ UTR extension. We observed FRET lifetimes consistent with photobleaching of Cy5 (Supplementary Fig. S10B). Substitution of ATP with its nonhydrolyzable analog ADPNP with all factors present also inhibited the rapid extension of DENV2 5′-3′ UTR conformation (Supplementary Fig. S10B–D). Instead, we observed occasional FRET-off states that persisted for just 0.4 s (95% CI: 0.3–0.4 s) before returning to more stable high FRET states (Supplementary Fig. S10D). Together, these results imply that eIF4A acts in concert with the other factors in an ATP-dependent manner to remodel vRNA conformation, likely through specific targeting to the 5′-m^7^G cap, but that these RBPs cannot maintain a stable, extended conformation without ATP hydrolysis by eIF4A.

## Discussion

### Orthoflavivirus vRNA folds into at least two unusually stable conformations

**Figure 7.**
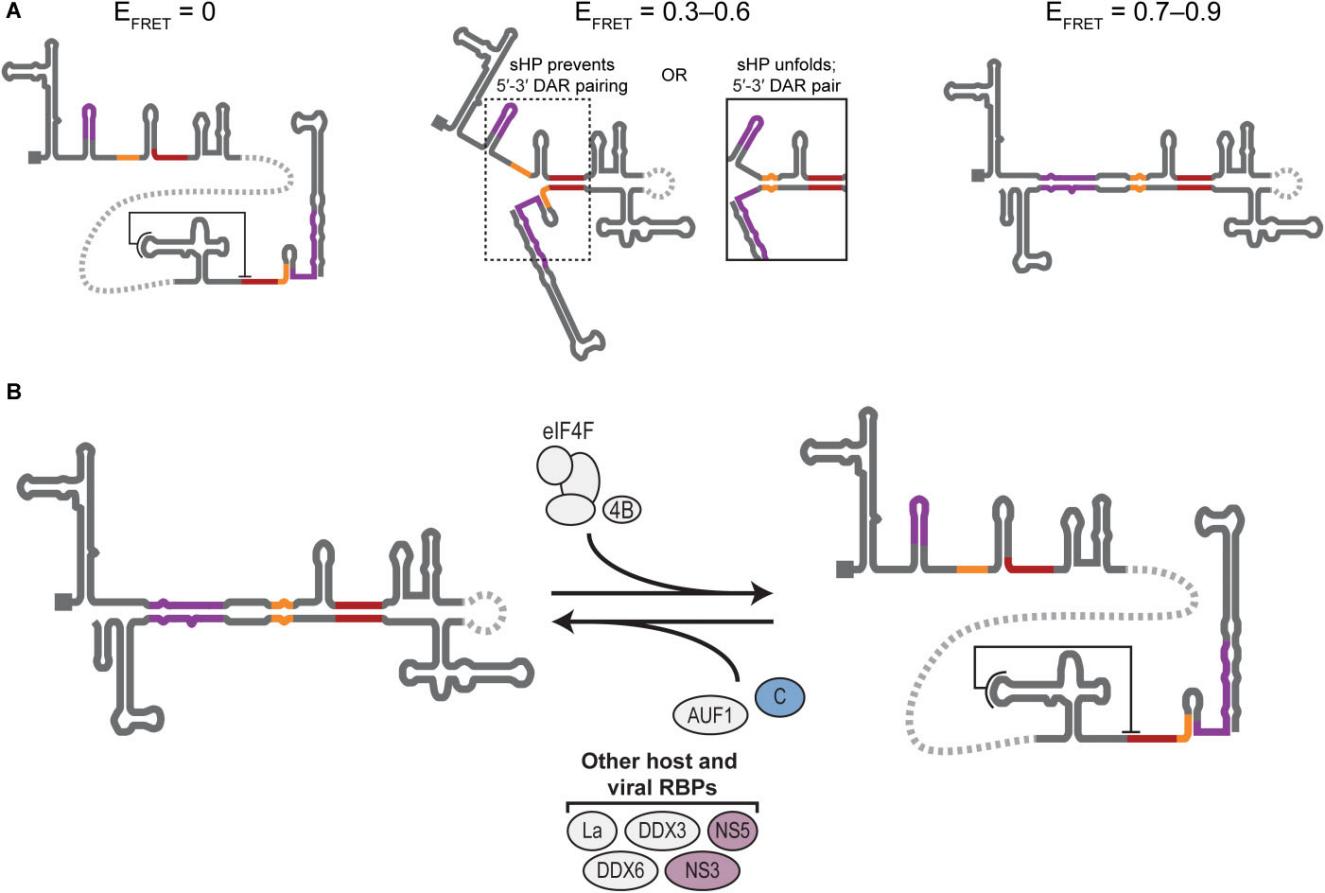
Model for orthoflaviviral conformations. (**A**) Proposed secondary structure conformations of distinct FRET states observed in this work. (**B**) Model for the essential role of host and viral RBPs in mediating the conformational switch between extended and cyclized states of vRNA. The slow exchange rate observed between extended and cyclized conformations may require active remodeling to enable *cis-*regulatory functions of the conserved complementary sequences during infection. RBPs that bind the terminal regions could modulate long-range base pairing or other tertiary contacts to disrupt the high stability of the extended and cyclized conformations observed with RNA alone in this study.

Orthoflaviviruses have conserved complementary sequences in the terminal regions of their genomes that are proposed to cyclize vRNA noncovalently to bring the 5′ and 3′ ends into proximity. We used smFRET between the 5′ and 3′ ends of vRNA minigenome constructs derived from representative orthoflaviviruses and full-length DENV2 vRNA to investigate their conformational ensembles. Using 5′-3′ FRET, we observed multiple conformational states (Fig. 7A). One conformation places the ends in close proximity (<4 nm), others yield modestly further apart ends (4–8 nm), and one or more states place the ends much further apart (>8 nm), outside the range of FRET. In representative orthoflaviviral sequences, the *E*_FRET_ ≈ 0.8 cyclized state and *E*_FRET_ = 0 extended state(s) were most prevalent in the ensemble. This distribution was observed regardless of the length of the RNA construct, which ranged from ∼750 to 11 000 nt.

The fraction of cyclized DENV2 vRNA decreased as sequence length increased. This is consistent with previous reports that the *in vitro* SHAPE reactivity profile of protein-free DENV2 vRNA predicted an extended vRNA conformation [35, 41]. Since SHAPE reactivity is ensemble averaged, signal for a minor state (16 ± 4% by smFRET) would be convoluted with that from the major population and hard to detect in these data. Our *in vitro* refolding in the absence of RBPs that interact with the vRNA throughout the viral infection cycle may allow it to access alternative states that prevent long-range interactions, decreasing the prevalence of cyclized vRNA in the population. These may include other long-range interactions that have been reported for full-length orthoflaviviral vRNAs during infection, such as pairing between the capsid and NS5 coding regions found in DENV and ZIKV [41] or between the 5′ UTR and Env coding region in ZIKV [37].

Proximity of the 5′ and 3′ ends is an intrinsic property of natural RNA sequences that has been experimentally verified for multiple mRNA, vRNA, and long noncoding RNA sequences [28–30]. We also detected 5′-3′ FRET in human β-globin mRNA, and obtained results comparable to previous measurements [30]. β-globin mRNA contains previously identified complementary sequences in the 5′ and 3′ UTRs whose interaction may stabilize the FRET-on states we observe [61, 62]. Importantly, these are short stems (3–5 bp) that are typical of secondary structure elements predicted computationally for most natural RNA sequences [63], which have rates of conformational transitions [64] comparable to the observed rates of FRET transitions [30]. All these data agree that typical RNA sequences transition between alternative secondary or tertiary FRET-on conformations on a timescale of seconds. In contrast, orthoflaviviral vRNA sequences showed long-range interactions that were significantly more stable. The long-range stems that stabilize FRET-on states are 11–15 bp. Model oligonucleotide duplexes of this length can have dissociation rates of 10^–2^ – 10^–10^ s^-1^ depending on GC content [65, 66]. Since measurement of the lifetimes of these states was limited by photobleaching of the fluorescent dyes (≥2.4 ± 0.3 minutes for DENV2 5′-3′ UTR at 37°C), this likely represents a lower bound on the intrinsic rate of interconversion between extended and cyclized 5′-3′ UTR.

### Cyclization depends on a multipartite interaction between the 5′ and 3′ terminal regions

Orthoflavivirus vRNAs with conserved complementary sequences have two or three regions of complementarity that are separated by other secondary structure elements. In the mosquito-borne clade, three complementary sequences, the UAR, DAR, and CS are essential for vRNA replication. Here, we identified that the unusual kinetic stability of the cyclized conformation of a representative vRNA sequence from this group was dependent on this multipartite interaction between the 5′ and 3′ terminal regions. Disruption of either the CS or UAR interaction in the DENV2 5′-3′ UTR could accelerate the rate of transitions between extended and cyclized states >200-fold (Fig. 4 and Supplementary Fig. S6).

Disrupting the 5′-3′ CS interaction made 5′-3′ FRET less frequent in the ensemble (∼10% of single-molecule traces, Fig. 4B and Supplementary Fig. S6B). Some CS mutants were observed to transiently sample a high FRET state (Supplementary Fig. S6C), possibly via brief but unsuccessful interaction of the 5′ and 3′ DAR or UAR sequences or other tertiary contacts. While compensatory mutations restoring 5′-3′ CS complementarity rescued folding into the high FRET state (Fig. 4B and Supplementary Fig. S6D), the mean *E*_FRET_ value was shifted relative to the WT DENV2 5′-3′ UTR. It is possible that the compensatory mutants enable alternative folding of the terminal regions that results in different proximity of the ends in the cyclized state, or that primary sequence effects on tertiary structure such as noncanonical base pairing or relative helix orientation at junctions propagates to subtle changes in the end-to-end distance. DENV2 vRNA with the CS rescue 1 sequence also has impaired replication relative to WT vRNA when transfected into mammalian or mosquito cells [21].

With complete disruption of the 5′-3′ UAR interaction by the DNA blocking oligonucleotide, we observed FRET transitions between a FRET-off state and intermediate FRET state (*E*_FRET_ = 0.29 ± 0.09) at a rate similar to conformational changes seen in β-globin and other mRNA sequences (Fig. 4C–E) [30]. These results suggest that the 5′-3′ CS and/or DAR interactions on their own are not sufficient to promote the long-lived high FRET state characteristic of the WT orthoflaviviral sequences (Fig. 7A). Importantly, disruption of the 5′ or 3′ UAR sequences by blocking oligonucleotide or mutations had little effect on the portion of the ensemble in a FRET-off state, even when they disrupted base pairing in SLB or 3′-SL (Supplementary Fig. S6F and G). This suggests that there are other structural elements responsible for the kinetic stability of the extended state(s), potentially including the 3′-DB pseudoknot (Supplementary Fig. S1C) [34, 67].

Intriguingly, the UAR mutants we tested had little effect on the 5′-3′ FRET efficiency distribution and conformational dynamics (Supplementary Fig. S6F and G). Both of these mutations were previously found to be deleterious for viral replication in cultured cells [14]. They may have subtle effects on the stability of the cyclized state that we do not observe during the timescale of our smFRET experiments (minutes). This could be sufficient to destabilize the 5′-3′ UAR interaction and inhibit cyclization in a cellular context, where host and viral RBPs interacting with the vRNA could facilitate structural rearrangements that inhibit replication by NS5. These mutations could also disrupt the tertiary structure of the vRNA terminal regions in a way that does not dramatically change the end-to-end distance as measured by 5′-3′ FRET but prevents essential conformational rearrangements or interactions with RBPs.

### Relative stabilities of local and long-range UAR interactions are not the only factors regulating cyclization

The regulatory function of the cyclization sequences and terminal vRNA secondary structures is proposed to require a balance of extended and cyclized vRNA, governed by the relative stabilities of competing long-range (5′-3′ UAR and DAR) and local (SLB and 3′-SL) base pairing [19]. Mutations that stabilize long-range interactions or weaken these local structures were both found to decrease viral replication in cultured cells [19, 25]. We observed that of all the orthoflaviviral sequences we tested, the DENV1 5′-3′ UTR had a strikingly different FRET distribution and conformational dynamics (Supplementary Fig. S7) that were instead similar to β-globin mRNA (Fig. 3D). We hypothesized that conserved sequence differences between these strains altered the stability of the local UAR structures leading to a preference for local over long-range base pairing. However, stabilizing SLB and 3′-SL in DENV2 and destabilizing these structures in DENV1 had little effect on 5′-3′ FRET in either. One possibility is that other RNA structural elements such as noncanonical base pairs or tertiary interactions in the DENV2 SLB and 3′-SL stabilize 5′-3′ UAR base pairing and cyclization in a way that these mutations stabilizing canonical pairing in the secondary structure cannot overcome. Similarly, such factors could compensate for weakened local structures in the DENV1 SLB/3′-SL mutant. Investigation of conserved sequence substitutions more distal to the cyclization sequences may help elucidate differences in RNA conformational dynamics between these strains.

### vRNA conformational stability may be necessary for *cis-*regulatory function in cells

Although 5′-3′ end proximity has been observed in a variety of natural RNA sequences, both previous measurements of the end-to-end distance and those presented here have been performed on RNAs refolded *in vitro* in the absence of proteins. Since RNAs interact with numerous RBPs including helicases as well as other RNAs (e.g. microRNAs), it is unclear how frequently intramolecular interactions will bring the 5′ and 3′ ends into close proximity in a cellular context. For mRNAs that are actively translated, the ribosome itself is a robust helicase capable of unfolding RNA structures like the short 4–5 bp stems predicted to be the most common element of mRNA secondary structure [63]. One study investigating the nature of mRNA conformation in the cytoplasm found that the ends of mRNA are quite distant from each other under normal conditions, and only under translation-inhibiting stress did mRNAs become compact with closer end-to-end distances as had been observed *in vitro* [68, 69]. The significantly greater kinetic stability of orthoflaviviral vRNAs conformations that we measured may reflect evolutionary pressure to maintain or refold a functional cyclized conformation when faced with active structural remodeling by translation and helicases. Since most known orthoflaviviruses have two hosts, any essential vRNA regulatory structures must robustly fold in the diverse cellular environments and especially the different temperatures at which these viruses need to replicate.

The long lifetimes of both extended and cyclized vRNA suggests that an extrinsic factor may be needed to trigger the conformational switch proposed to facilitate their regulatory function. Our results show that the human translation initiation factors eIF4A, eIF4B, eIF4E, and eIF4G collectively facilitate remodeling of cyclized vRNA into an extended conformation (Fig. 6 and Supplementary Fig. S10). Remodeling depended on ATP hydrolysis and likely targeting of the eIF4F complex to the 5′-m^7^G cap, as the DEAD-box helicase eIF4A was incapable of promoting conformational rearrangements in the absence of the other factors. While eIF4A readily associates with RNA in the absence of other factors [70], association with eIF4E, eIF4G, and eIF4B greatly enhances its processivity as a helicase [53, 71]. A recent study has shown that yeast eIF4F can bind nonspecifically across the entire RNA before its stable association with the 5′ end [72], but it is unknown whether it modulates RNA structure during these transient binding events. Future work on this early step of translation initiation may provide insight into whether other vRNA structural elements, such as regulatory RNA folds in the ORF [38, 40], are also remodeled by eIF4F.

These experiments provide mechanistic insight into how the compact, cyclized conformation of vRNA that is packaged in the virion may be remodeled upon entry into a new host cell. Since translation by host ribosomes is the first role of vRNA during infection, factor-mediated refolding of vRNA into an extended conformation that can be accommodated in the 40S ribosomal mRNA entry channel may be a crucial first interaction of the vRNA with host RBPs. Other host RBPs have been identified in both human and mosquito cells that are beneficial to viral fitness and interact with the terminal regions of vRNA [73–83]. AU-rich element RNA-binding protein 1 (AUF1) has been reported to accelerate displacement of the 3′-SL for interaction with an oligonucleotide corresponding to the 5′-UAR sequence [78, 80]. The viral capsid protein is also reported to have a similar biochemical activity on model substrates [77]. Future work assessing whether these factors preferentially stabilize certain vRNA conformations should help elucidate their biological function during infection (Fig. 7B).

By developing a tool to track vRNA conformation in real-time, we have shown direct evidence of intramolecular cyclization by long-range interactions in full-length orthoflaviviral vRNA. Monitoring vRNA conformation during viral translation, genome replication, and packaging will help determine the detailed mechanisms by which alternative vRNA conformations regulate these biological processes.

## Supplementary Material

gkaf514_Supplemental_Files

## Data Availability

The data underlying this article will be shared on request to the corresponding author.

## Notes

Present address: Department of Biochemistry and Molecular Genetics, University of Colorado Anschutz Medical Campus, Aurora, CO 80045, United States
