# Supplementary material for: Conserved long-range interactions are required for stable folding of orthoflaviviral genomic RNA
Source: Nucleic Acids Res. 2025 Jun 16;53(11):gkaf514. doi: 10.1093/nar/gkaf514 (PMC12168079; doi:10.1093/nar/gkaf514)
Supplement: gkaf514_Supplemental_Files [file gkaf514_supplemental_files.zip › Supplementary_data_revised.pdf]

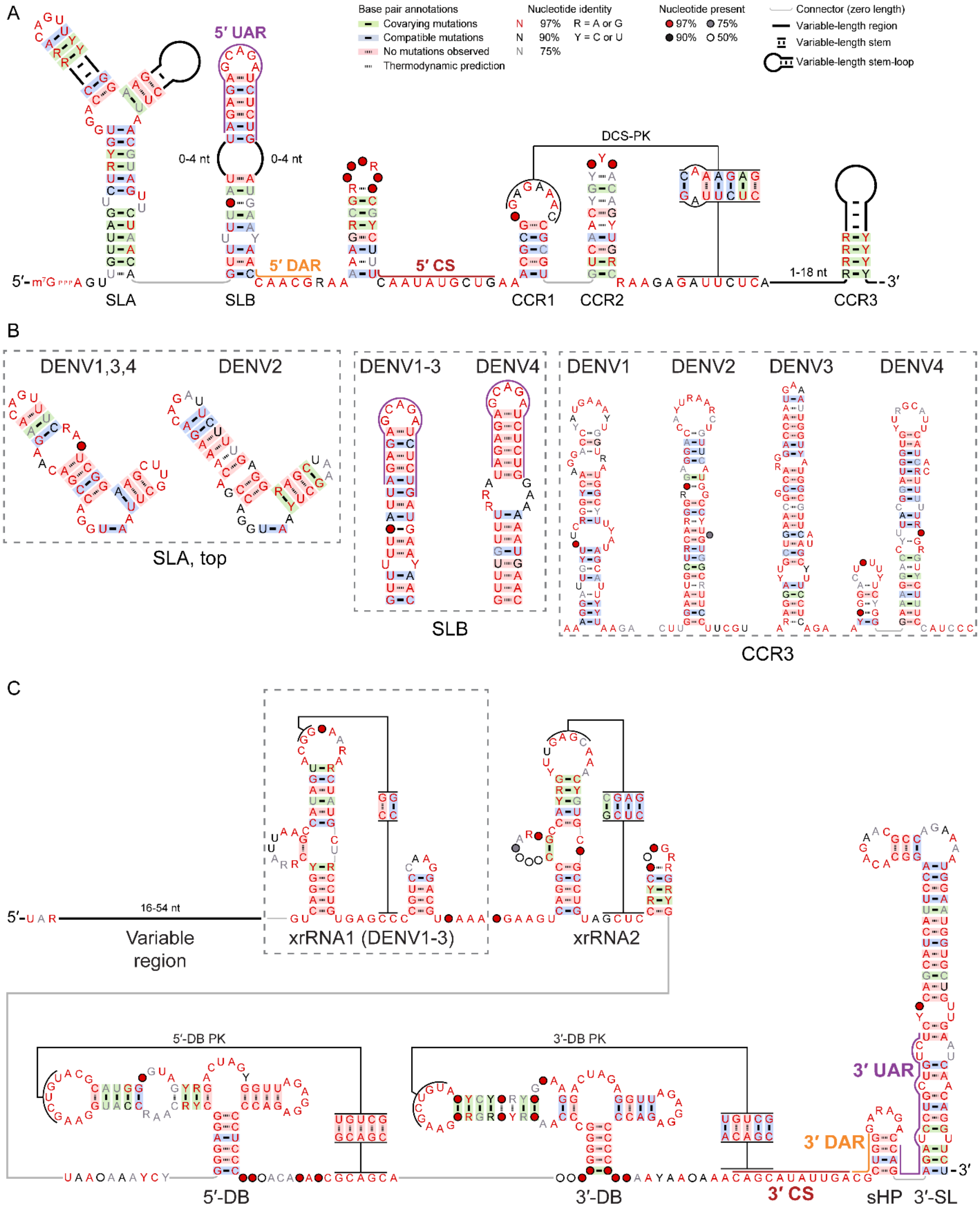

Fig. S1 Bioinformatic analysis of full DENV terminal regions. (**A**) Sequence-structure conservation diagram of the 5′ terminal region of DENV vRNA. Sequence conservation and base pair covariation for 1418 sequences were analyzed and diagram prepared with R2R (1). Base

pairs supported by phylogenetic analysis are shown with a solid line, and base pairs that are only supported by secondary structure prediction are indicated with a dashed line. Evolutionarily supported base pairs were annotated as covarying and highlighted in green if both nucleotides changed identities in one or more strains (e.g. G-C to A-U), and were annotated as compatible and highlighted in blue if just one residue changed (e.g. G-C to G-U). Predicted base pairs between universally conserved nucleotides are highlighted with a red box. The font color of individual residues indicates primary sequence conservation. R and Y stand for positions that must be purines or pyrimidines, respectively. Filled circles indicate residues that are absent (gap in the MSA) in the indicated fraction of DENV sequences analyzed. The first start codon does not appear to be well conserved because its position within the lower portion of SLB varies between serotypes. (**B**) Modular secondary structures from panel **a** with different base pairing in the indicated serotypes. (**C**) Sequence-structure conservation diagram of the full 3′ UTR of DENV vRNA, annotated as in panel **A**. xrRNA 1 is absent in DENV4 sequences.

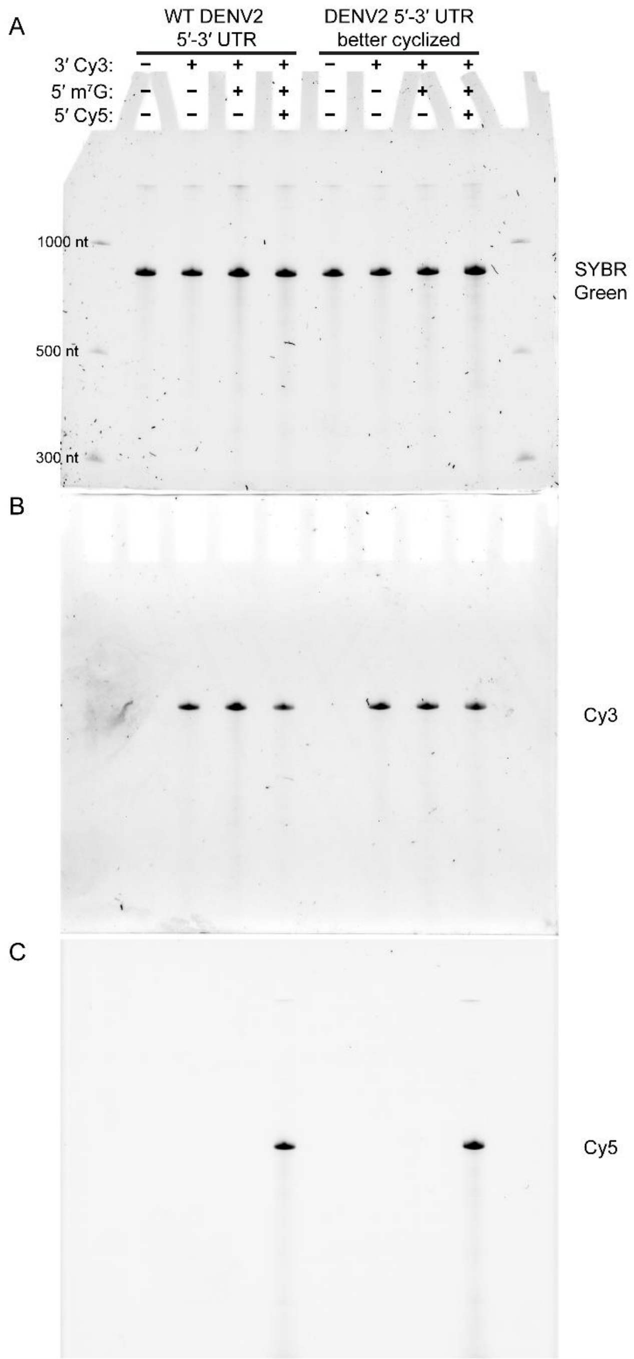

Fig. S2 Denaturing PAGE analysis of doubly labeled 5′-3′ UTR RNAs. (**A**) 5% polyacrylamide-TBE denaturing gels were run as described in **Methods**. 100 ng RNA was loaded in each gel lane. NEB Low Range ssRNA ladder was loaded on each side of the gel. Total RNA present in intermediate products and final doubly labeled RNAs was visualized by staining with SYBR Green II. (**B**) Same gel as above, scanned for Cy3 fluorescence before SYBR Green II staining. (**C**) Same gel as above, scanned for Cy5 fluorescence before SYBR Green II staining.

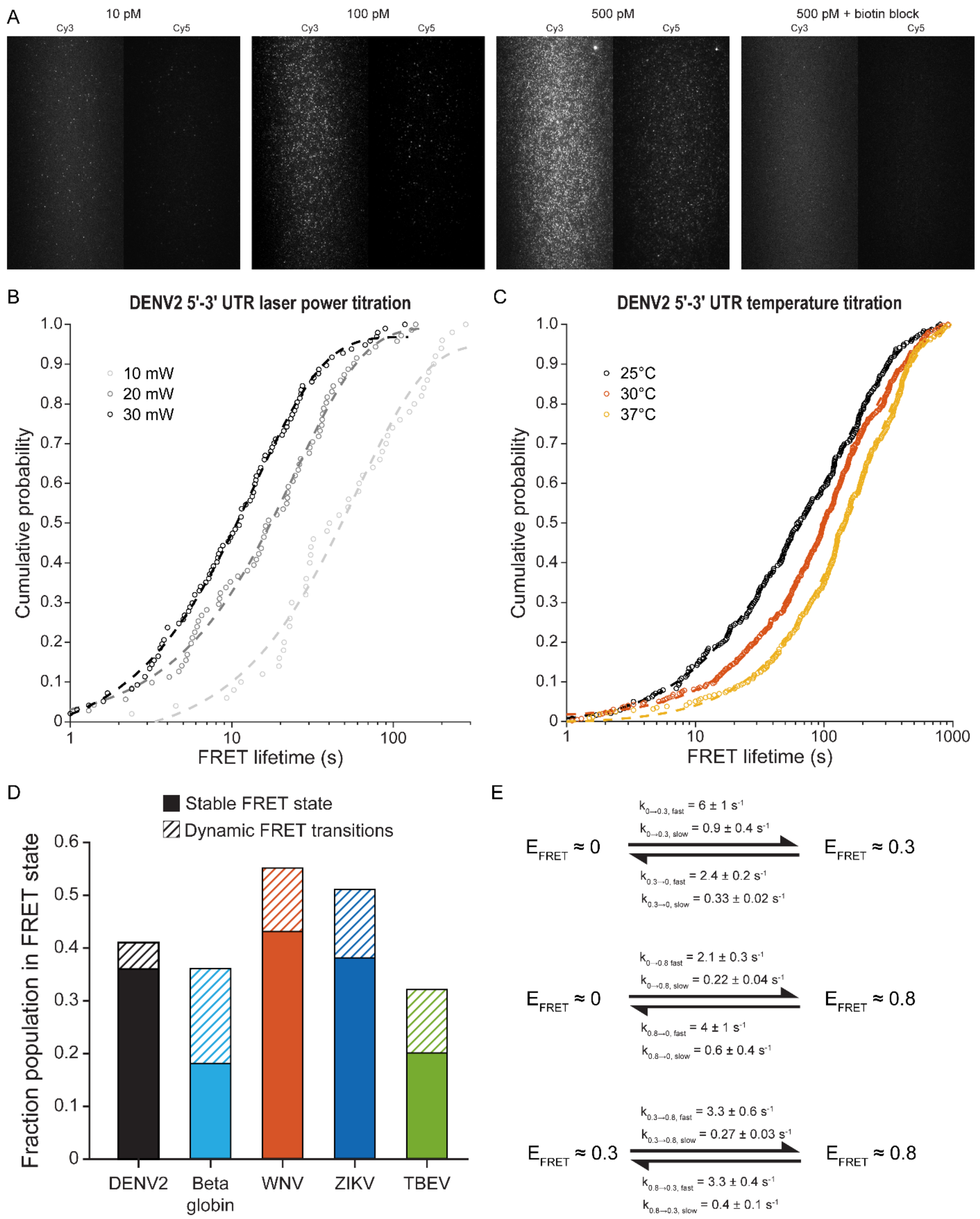

Fig. S3 Additional data related to Fig. 3 (**A**) RNA immobilization for TIRF microscopy is concentration- and biotin-dependent. Varying concentrations of DENV2 5′-3′ UTR in complex with biotinylated tethering oligo were immobilized on passivated and neutravidin-coated quartz

slides for 5 min at room temperature. For the biotin block, the imaging surface was incubated with 1 mM biotin for 5 min and washed twice before RNA immobilization. Representative fields of view illuminated with a 532 nm laser at 30 mW total power are shown. Contrast is 1,000-10,000 counts for all images. (**B**) DENV2 5′-3′ UTR FRET lifetime is limited by photobleaching of Cy5. Observed FRET lifetime increases with decreasing laser power. Fields of view illuminated with 20 or 30 mW total power were imaged for 3 min, while those illuminated with 10 mW were imaged for 5 min. (**C**) DENV2 5′-3′ UTR is stable at physiological temperatures. DENV2 5′-3′ UTR smFRET was observed on a custom Pacific Biosciences RSII as described in **Methods**. The increase in observed FRET lifetime seen at higher temperatures is likely due to lower oxygen solubility and/or increased activity of the oxygen scavenging system. (**D**) Quantification of different FRET populations observed in dual illumination experiments for orthoflaviviral 5′-3′ UTR RNAs and β-globin mRNA. n = 309 traces were analyzed for DENV2 5′-3′ UTR, n = 306 for β-globin mRNA, n = 163 for WNV 5′-3′ UTR, n = 397 for ZIKV 5′-3′ UTR, and n = 161 for TBEV 5′-3′ UTR. (**E**) Observed rate constants for transitions between 5′-3′ FRET states of β-globin mRNA. Transition times between states were fit to double exponential equations to extract rate constants. The error reported represents the 95% confidence interval (C.I.) of the fit.

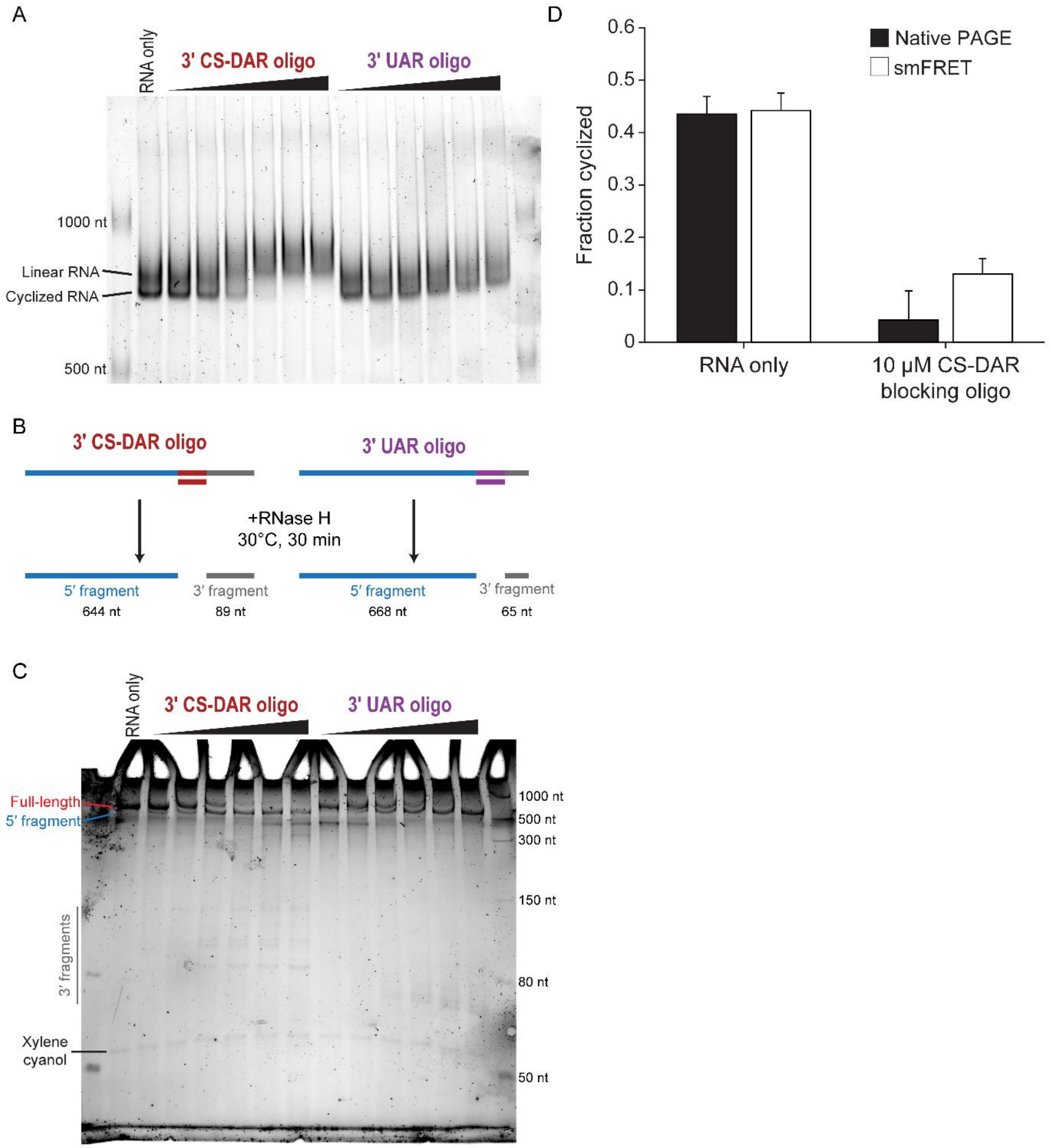

Fig. S4 DENV2 5′-3′ UTR folds into at least two conformations that depend on long-range interactions. (**A**) Native PAGE analysis of DENV2 5′-3′ UTR conformation. Oligo to RNA molar ratios ranged from 0.5 to 100. Gel was run as described in **Methods**, stained with SYBR Green II, and visualized on a UV transilluminator. The apparent shift upward of RNA bands in the UAR oligo titration series is due to uneven running of the gel rather than a change in electrophoretic mobility, based on the position of the ssRNA ladder bands. (**B**) Schematic of the

approach to validate DNA blocking oligo binding using RNase H digestion. (**C**) RNase H digestion of the samples analyzed by native PAGE in panel **A**. 100 ng RNA was loaded in each gel lane. NEB Low Range ssRNA ladder was loaded on each side of the gel. The locations of different RNA species are indicated on the left side. The band migrating slightly above 50 nt in all samples is xylene cyanol from the loading dye. Gels were visualized by staining with SYBR Green II. (**D**) Quantification of native PAGE results and comparison with results obtained by smFRET. The fraction of total intensity contained in the lower band was quantified by densitometry analysis. Error bars represent the standard deviation of n=3 independent experiments. Results from dual illumination TIRF microscopy analysis are derived from the data presented in **Fig. S5B**, **Fig. S6B**, and additional experiments. Error bars for the RNA only condition represent the standard deviation of n=4 independent experiments. Error bars for the 10 μM CS-DAR blocking oligo condition represent the 95% C.I. from the data presented in **Fig. S6B**.

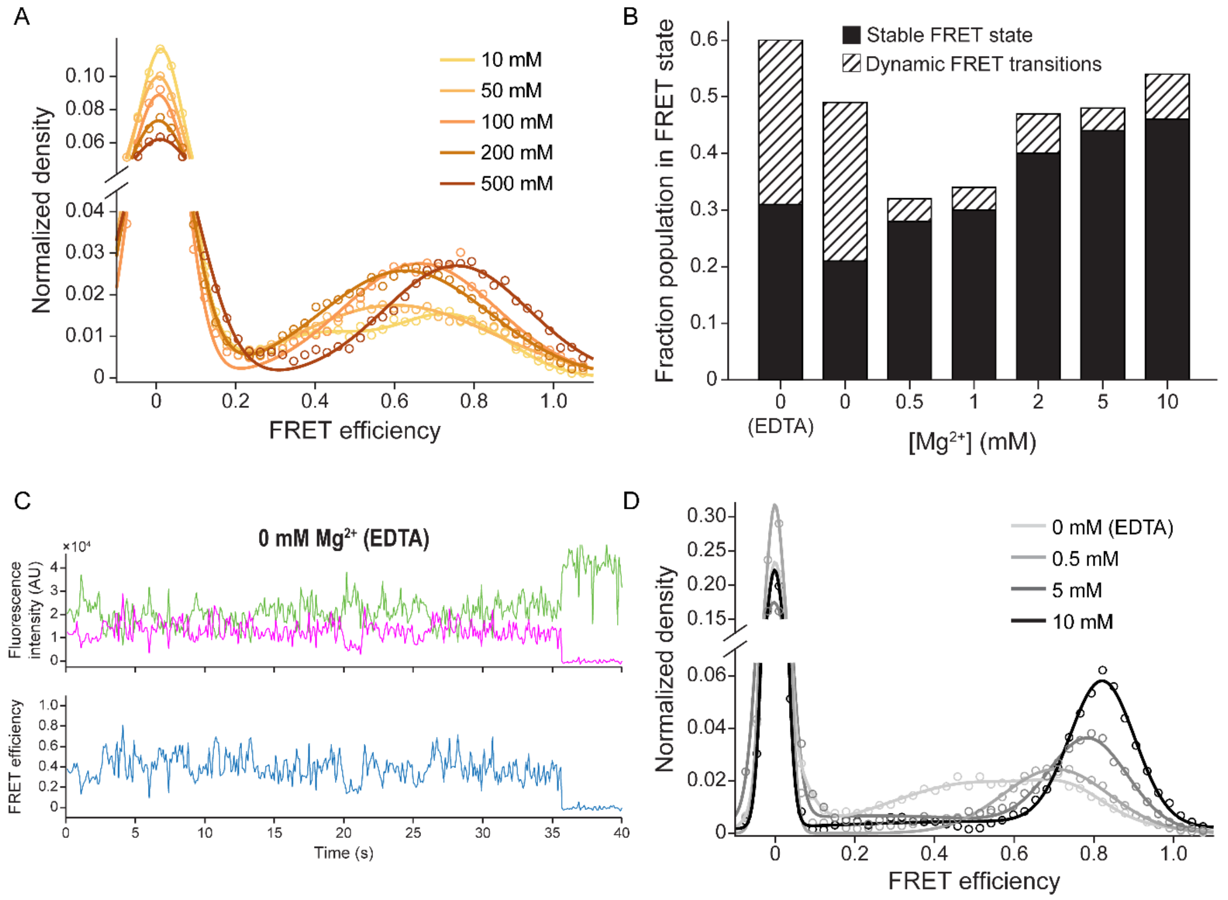

Fig. S5 DENV2 5′-3′ UTR stability depends on Mg^{2+}. (**A**) FRET efficiency distributions of WT DENV2 5′-3′ UTR refolded and imaged with different monovalent ion concentrations. The solid lines are the Gaussian fit to the observed data. Number of single illumination and dual illumination traces analyzed for each construct, respectively, were n=190 and n=714 for 10 mM K^+, n=163 and n=678 for 50 mM K^+, n=82 and n=230 for 100 mM K^+, n=181 and n=763 for 200 mM K^+, and n=109 and n=634 for 500 mM K^+. The apparent decrease in the frequency of the E_{FRET} = 0 peak for the 200 mM and 500 mM K^+ conditions relative to 100 mM K^+ is due to peak broadening caused by decreased Cy3 signal-to-noise. (**B**) Quantification of different FRET populations observed in dual illumination experiments for DENV2 5′-3′ UTR after refolding and imaging at a range of Mg^{2+} concentrations. n=431 dual illumination traces were analyzed for 5 mM EDTA, n=313 for 0 mM Mg^{2+}, n=199 for 0.5 mM Mg^{2+}, n=470 for 1 mM Mg^{2+}, n=158 for 2 mM Mg^{2+}, n=230 for 5 mM Mg^{2+}, and n=336 for 10 mM Mg^{2+}. (**C**) Representative 5′-3′ smFRET trace for the DENV2 5′-3′ UTR refolded and imaged in the absence of Mg^{2+}. Top, Cy3

and Cy5 fluorescence intensity; bottom, FRET efficiency. To highlight rapid FRET state transitions, the trace is zoomed in on the region before Cy5 photobleaching and Cy3 photobleaching is not shown. (**D**) FRET efficiency distributions of selected Mg^{2+} concentrations. The solid lines are the Gaussian fit to the observed data. Number of single illumination and dual illumination traces analyzed for each construct, respectively, were n=313 and n=171 for 0 mM Mg^{2+}, n=199 and n=171 for 0.5 mM Mg^{2+}, n=114 and n=309 for 5 mM Mg^{2+}, and n=336 and n=191 for 10 mM Mg^{2+}.

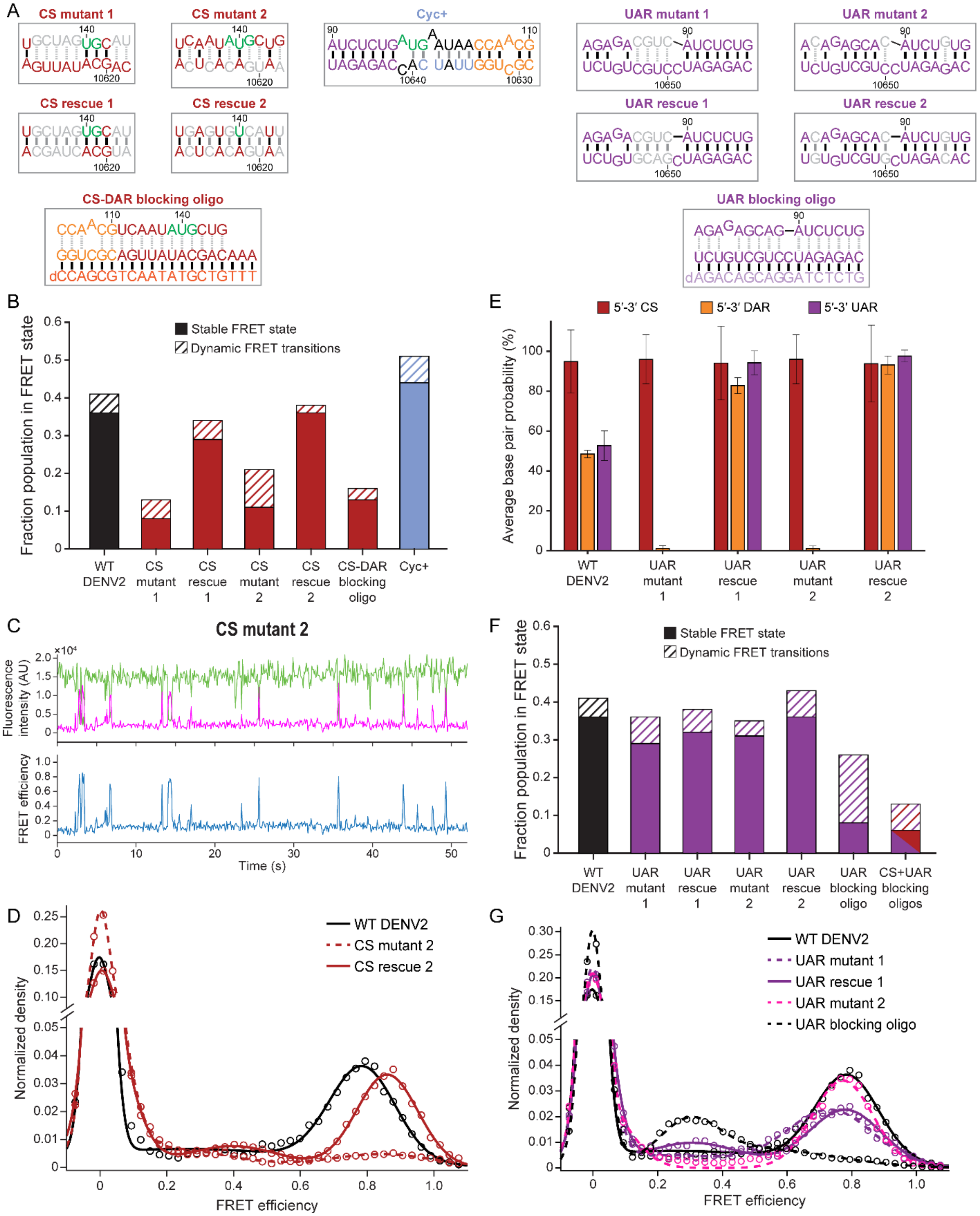

Fig. S6 Additional data related to Fig. 4. (**A**) DENV2 5′-3′ UTR variants and blocking oligonucleotides analyzed by smFRET. Sequence mutations are colored in gray and disrupted base pairs are indicated with a dashed gray line. Reconstituted base pairs in rescue mutants are

indicated with a solid gray line. Start codons are highlighted with green font. For the DNA blocking oligonucleotides, the 3′ CS-DAR blocking oligonucleotide is colored in orange and the 3′ UAR blocking oligonucleotide is colored in light purple. (**B**) Quantification of different FRET populations observed in dual illumination experiments for CS and Cyc+ variants. n = 309 traces were analyzed for WT, n = 359 for CS mutant 1, n = 314 for CS rescue 1, n = 276 for CS mutant 2, n = 302 for CS rescue 2, n = 476 for CS-DAR blocking oligonucleotide, and n = 263 for Cyc+. (**C**) Representative 5′-3′ smFRET trace for the CS mutant 2 5′-3′ UTR. Top, Cy3 and Cy5 fluorescence intensity; bottom, FRET efficiency. To highlight rapid FRET state transitions, the trace is zoomed in on the region before Cy5 photobleaching and Cy3 photobleaching is not shown. (**D**) FRET efficiency distributions of DENV2 5′-3′ UTR CS mutants not shown in **Fig. 4B** are plotted with open circles. The solid lines are the Gaussian fit to the observed data. Number of single illumination and dual illumination traces analyzed for each construct, respectively, were n=114 and n=309 for WT, n=74 and n=276 for CS mutant 2, and n=181 and n=302 for CS rescue 2. (**E**) Mean base pairing probabilities for 5′-3′ CS, DAR, and UAR in different WT 5′-3′ UTR constructs. Error bars are the standard deviation of probabilities observed for the individual base pairs in the interaction (n = 11 for CS, n = 5 for DAR, and n = 15 for UAR). Base pairing probabilities were calculated from the partition function predicted for each sequence using RNAstructure. (**F**) Quantification of different FRET populations observed in dual illumination experiments for UAR variants. n = 309 dual illumination traces were analyzed for WT, n = 290 for UAR mutant 1, n = 525 for UAR rescue 1, n = 648 for UAR mutant 2, n = 272 for UAR rescue 2, n = 675 for UAR blocking oligonucleotide, and n = 1136 for CS/DAR + UAR blocking oligonucleotides. (**G**) FRET efficiency distributions of DENV2 5′-3′ UTR UAR mutants are plotted with open circles. The solid lines are the Gaussian fit to the observed data. Number of single illumination and dual illumination traces analyzed for each construct, respectively, were n=114 and n=309 for WT DENV2 5′-3′ UTR, n=199 and n=290 for UAR mutant 1 5′-3′ UTR, n=203 and n=525 for UAR rescue 1 5′-3′ UTR, n=289 and n=648 for UAR mutant 2 5′-3′ UTR, and n=525 and n=187 for UAR blocking oligonucleotide.

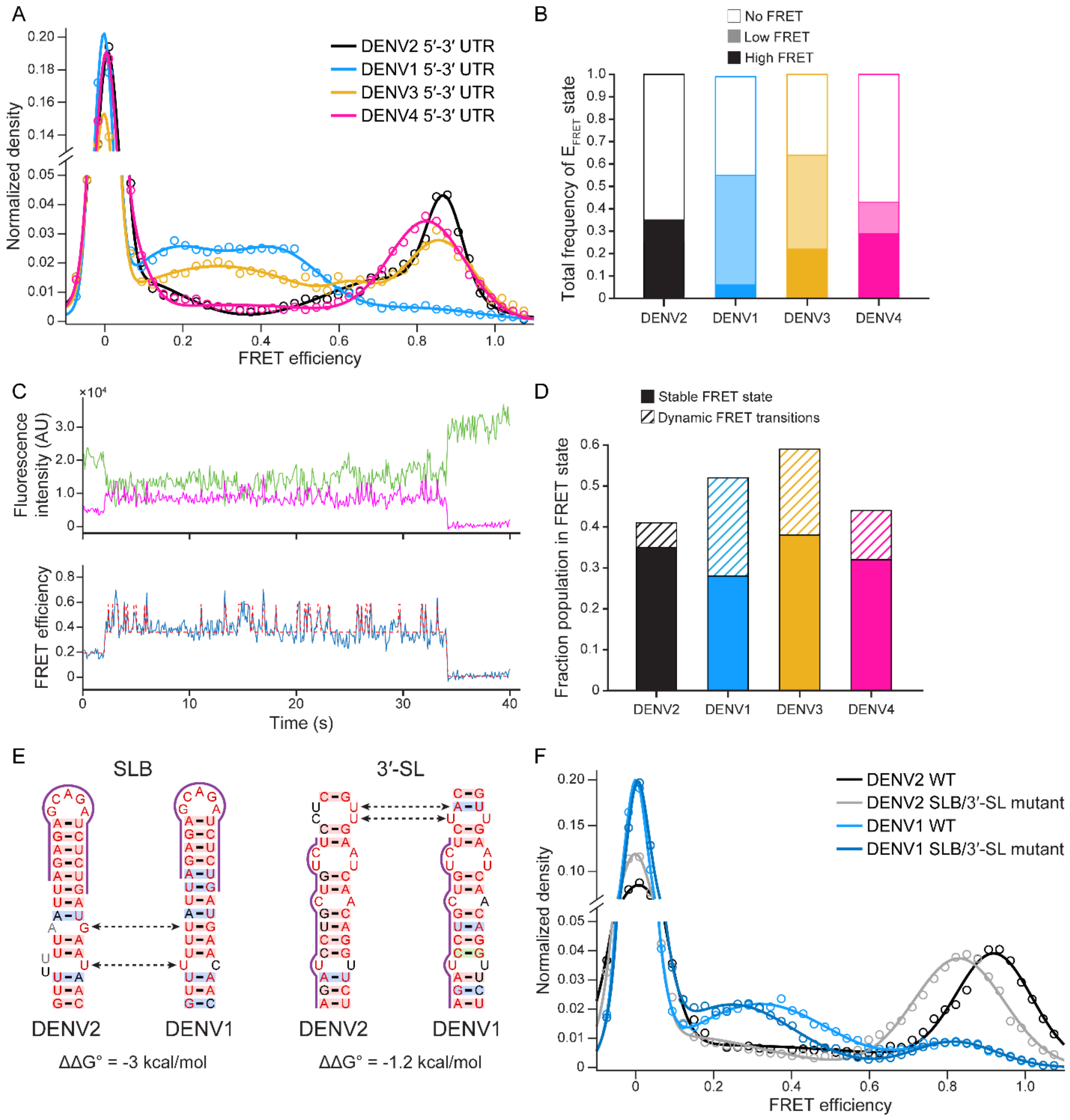

Fig. S7 Closely related DENV serotypes have distinct conformational ensembles. (**A**) FRET efficiency distribution of representative 5′-3′ UTR sequences for each serotype. Number of single illumination and dual illumination traces analyzed for each construct, respectively, were n=114 and n=309 for DENV2 5′-3′ UTR, n=151 and n=143 for DENV1 5′-3′ UTR, n=240 and n=353 for DENV4 5′-3′ UTR, and n=345 and n=433 for DENV4 5′-3′ UTR. The solid lines are the Gaussian fit to the observed data. (**B**) Frequency of different FRET states in representative

DENV vRNAs. Individual peaks that fit a Gaussian distribution were binned as no FRET if $\mu < 0.1$, low FRET for $0.1 < \mu < 0.6$, and high FRET for $\mu > 0.6$. Total frequency was calculated by integrating the parametrized Gaussian functions in each bin. (**C**) Representative 5′-3′ smFRET trace for the DENV1 5′-3′ UTR. Top, Cy3 and Cy5 fluorescence intensity; bottom, FRET efficiency. Dashed lines represent a four-state hidden Markov model fit to the observed FRET efficiency data. To highlight rapid FRET state transitions, the trace is zoomed in on the region before Cy5 photobleaching and Cy3 photobleaching is not shown. (**D**) Quantification of different FRET populations observed in dual illumination experiments for DENV serotype 5′-3′ UTRs. n = 309 traces were analyzed for DENV2 5′-3′ UTR, n = 143 for DENV1 5′-3′ UTR, n = 353 for DENV3 5′-3′ UTR, and n = 433 for DENV4 5′-3′ UTR. (**E**) Conserved structural differences between DENV1 and DENV2 terminal regions. Sequence-structure conservation models of SLB and the lower portion of 3′-SL were prepared as in **Fig. 1B-C** by analysis of 491 and 585 sequences for DENV1 and DENV2, respectively. Conserved differences between the serotypes are indicated with arrows. (**F**) FRET efficiency distribution of DENV1 and DENV2 5′-3′ UTR variants. SLB- and 3′-SL-strengthening mutations (ΔA77, U102C, C690U, and U691A) were introduced in DENV2 and SLB- and 3′-SL-weakening mutations (A76 insertion, C100U, U700C, A701U) in DENV1 5′-3′ UTR. Number of single illumination and dual illumination traces analyzed for each construct, respectively, were n=152 and n=230 for WT DENV2 5′-3′ UTR, n=223 and n=294 for DENV2 SLB/3′-SL mutant 5′-3′ UTR, n=197 and n=263 for WT DENV1 5′-3′ UTR, and n=207 and n=364 for DENV1 SLB/3′-SL mutant 5′-3′ UTR.

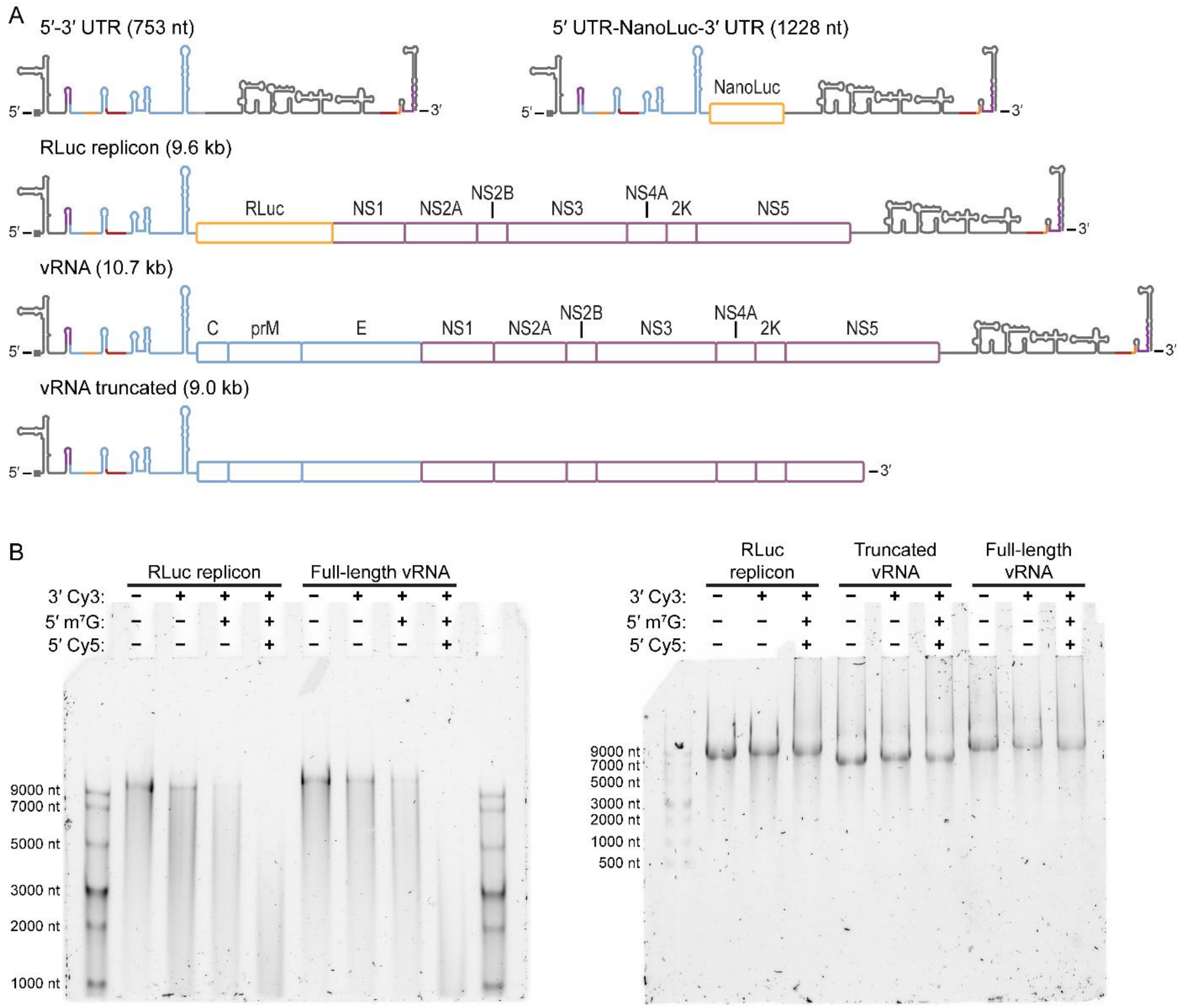

Fig. S8 Preparation of full-length DENV2 vRNA constructs. (**A**) Different length DENV2 constructs analyzed by 5′-3′ smFRET. ORFs are shown as boxes proportional to the length of the sequence. Polyprotein ORFs in the RLuc replicon and vRNA are subdivided into the protein products produced by proteolytic cleavage during infection. Terminal regions are shown as cartoon structures, not to scale with ORFs. (**B**) Denaturing agarose gel electrophoresis of preparation of double labeled vRNA constructs before and after protocol optimization. 500 ng RNA was loaded in each gel lane. NEB ssRNA ladder was loaded on each side of the gel as a size standard. Gels were visualized by staining with SYBR Green II.

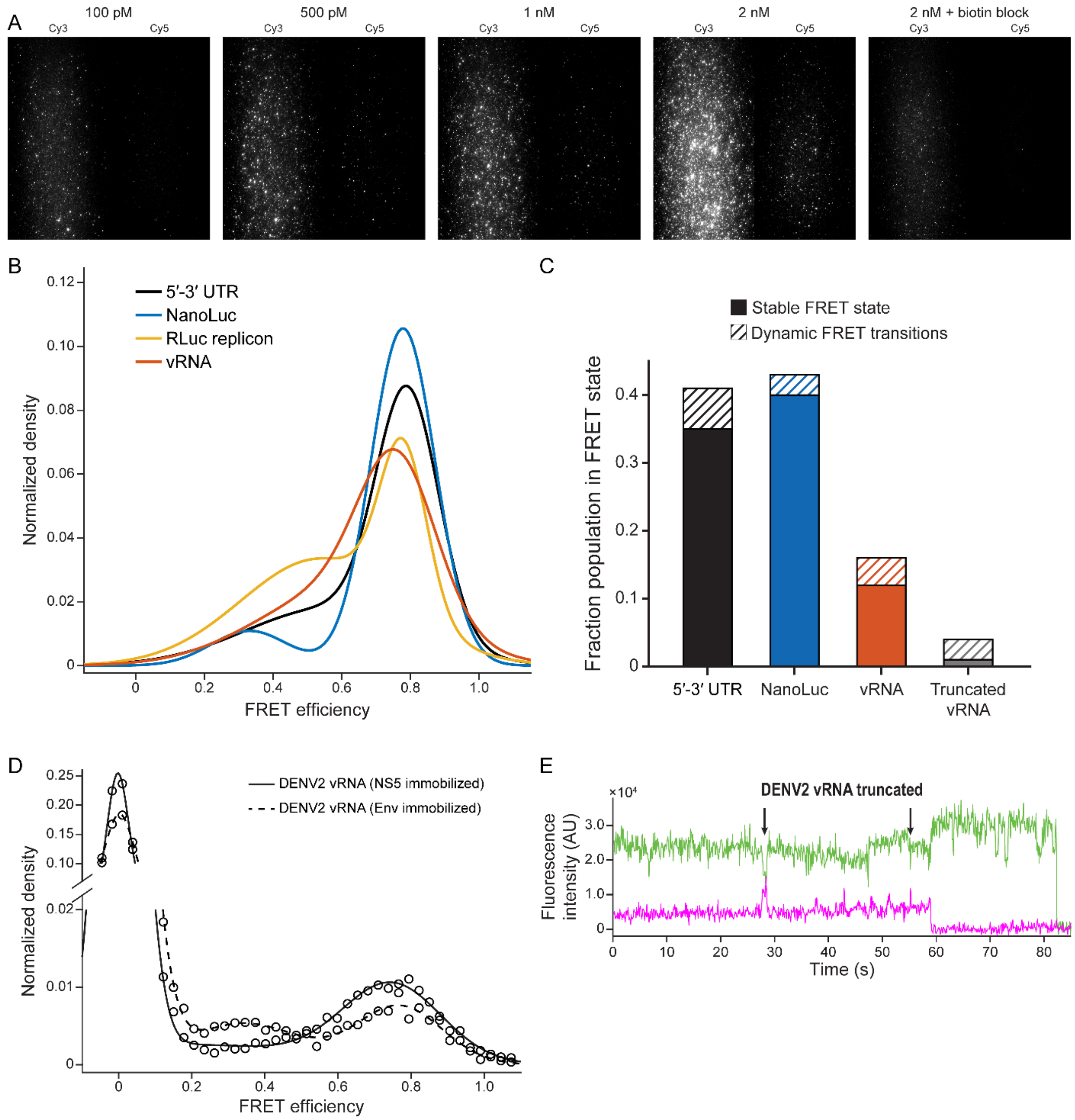

Fig. S9 Additional data related to Fig. 5. (**A**) vRNA immobilization for TIRF microscopy is concentration- and biotin-dependent. (**B**) DENV2 constructs share their major FRET state regardless of length. FRET efficiency distributions of DENV2 constructs are shown without the $E_{FRET} = 0$ peak. n=114 single illumination traces were analyzed for 5′-3′ UTR, n=292 for NanoLuc, n=147 for RLuc replicon, and n=67 for strain 16681 vRNA. (**C**) Quantification of different FRET populations observed in dual illumination experiments for different length DENV2 constructs. n=309 dual illumination traces were analyzed for 5′-3′ UTR, n=569 for

NanoLuc, n=423 for strain 16681 vRNA, and n=350 for truncated vRNA. (**D**) FRET efficiency distributions of alternative DENV2 vRNA immobilization sites. The solid lines are the Gaussian fit to the observed data. n=90 single illumination and n=423 dual illumination traces were analyzed for NS5-immobilized DENV2 vRNA and n=67 single illumination and n=323 dual illumination traces were analyzed for Env-immobilized DENV2 vRNA. (**E**) Representative 5′-3′ smFRET trace for the truncated DENV2 vRNA. Arrows indicate FRET transitions.

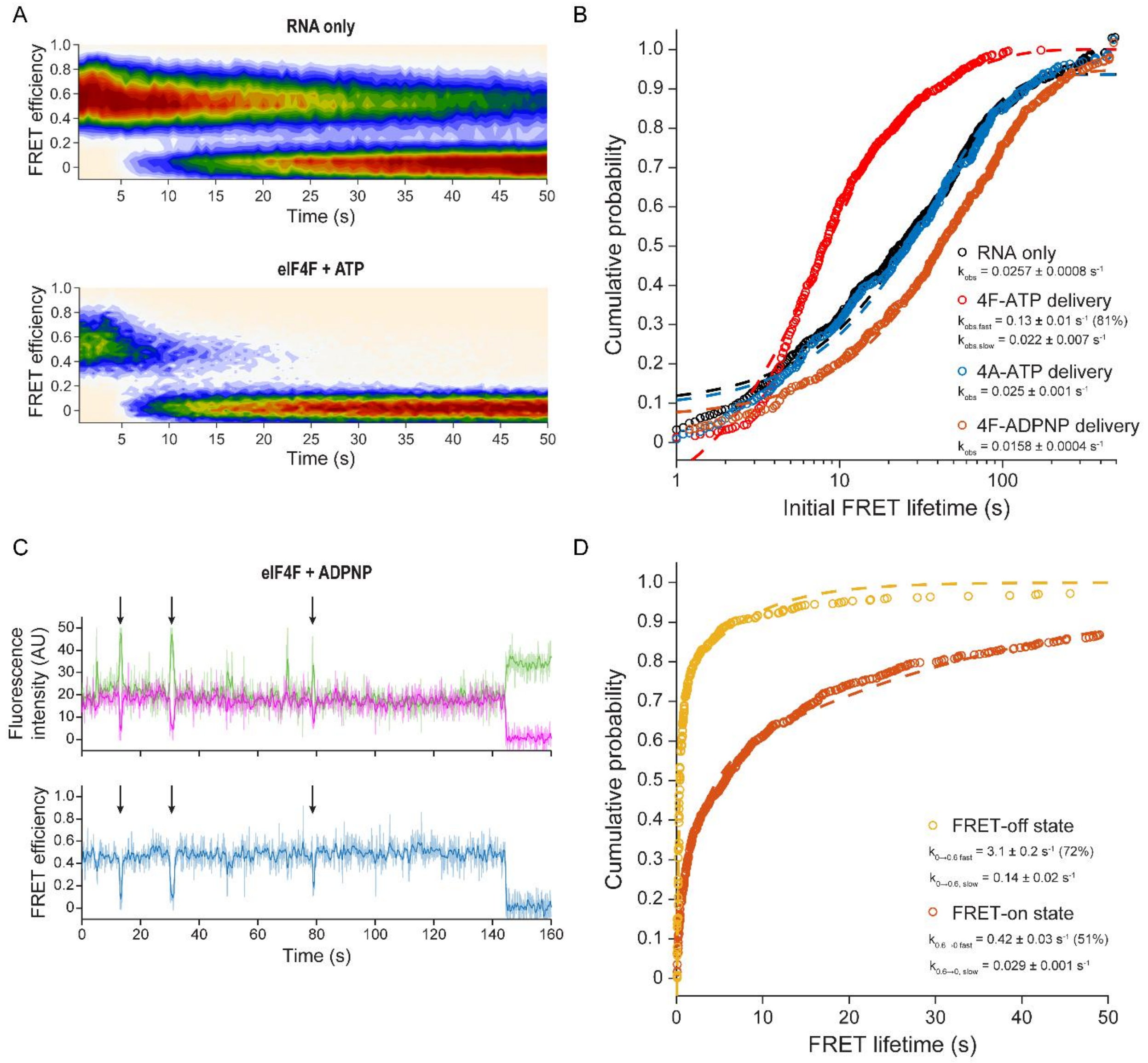

Fig. S10 Additional data related to Fig. 6. (**A**) Density maps of normalized doubly-labeled DENV2 5′-3′ UTR FRET efficiency after buffer or factor delivery for traces quantified in panel **B**. Buffer or factors were delivered at t=0 s. (**B**) Initial FRET lifetime observed after delivery of either buffer or eIF4 proteins. The number of traces analyzed were n=545 traces for RNA only, n=516 for eIF4F + ATP delivery, n=324 for eIF4A + ATP delivery, and n=355 for eIF4F + ADPNP delivery. The data were fit to single or double exponential equations to extract rate constants. A minor slow phase (19%) in the eIF4F + ATP delivery experiment has a rate of FRET loss consistent with photobleaching of Cy5. (**C**) Representative 5′-3′ smFRET trace for DENV2 5′-3′ UTR after delivery of eIF4F with 1 mM ADPNP. Factors were delivered at t=0 s.

The solid lines are fluorescence counts smoothed with a sliding window over n=5 frames. Raw data is displayed in light coloring. Arrows indicate FRET transitions. (**D**) Lifetimes of dynamic refolding events observed after delivery of eIF4F + ADPNP. n=614 transitions from FRET-on to FRET-off states and n=581 transitions from FRET-off to FRET-on states were analyzed from n=389 traces. The data were fit to double exponential equations to extract rate constants.

Table S1. RNA and DNA oligonucleotides used in this study.

Oligonucleotides (5′→3′)	
Biotin 5′-3′ UTR tethering oligo	rArGrGrCrCrGrGrGrCrCrUrUrUrCrUrUrUrArUrGrUrCrCrUrCrUrUrC/3Bio/
Biotin β-globin mRNA tethering oligo	rGrArCrCrArGrCrArCrGrUrUrGrCrCrCrArCrCrUrCrGrGrArC/3Bio/
Biotin NanoLuc tethering oligo	rGrCrArGrGrArUrCrArCrCrUrUrArArArGrUrGrArUrGrArUrCrUrArGrGrUrGrUrC/3Bio/
Biotin DENV2 strain 16681 Env tethering oligo	rCrCrUrUrGrCrCrArUrGrUrUrUrCrCrUrGrUrGrArGrUrArArArGrG/3Bio/
Biotin DENV2 strain 16681 NS5 tethering oligo	rUrUrCrUrUrGrCrUrCrArCrGrUrCrUrCrUrUrArGrArArUrGrUrUrUrGrGrArUrCrG/3Bio/
DENV2 CS-DAR blocking oligo	CCAGCGTCAATATGCTGTTT
DENV2 UAR blocking oligo	AGACAGCAGGATCTCTGGTC
DENV2 5′-3′ UTR Cyc+ forward primer	AAAAAACAGCATATTGACGCTGGTTATCACCAGAGATCCTGCTGTCTCCTC
DENV2 5′-3′ UTR Cyc+ reverse primer	GAGGAGACAGCAGGATCTCTGGTGATAACCAGCGTCAATATGCTGTTTTTT
DENV2 5′-3′ UTR UAR rescue 1 forward primer	GCTGGGAAAGACCAGAGATCGACGTGTCTCCTCAGCATCATTCC
DENV2 5′-3′ UTR UAR rescue 1 reverse primer	GGAATGATGCTGAGGAGACACGTCGATCTCTGGTCTTTCCCAGC
DENV2 5′-3′ UTR UAR rescue 2 forward primer	GCTGGGAAAGACCACAGATCGTGCTGTGTCCTCAGCATC
DENV2 5′-3′ UTR UAR rescue 2 reverse primer	GATGCTGAGGACACAGCACGATCTGTGGTCTTTCCCAGC

Table S2. dsDNA fragments encoding T7 transcription templates for RNA constructs. Flanking pUC19 plasmid sequence used for plasmid assembly are in lower case font. T7 promoter sequence and XbaI restriction site are underlined. The XbaI cut site is indicated with a vertical line. Linker sequences for 5′-3′ UTR constructs are in italics.

**DNA templates**	
DENV2 5′-3′ UTR	tgaccatgattacgccaagcttgcatgcctgcaggtcgacTAATACGACTCACT<br>ATTAGTTGTTAGTCTACGTGGACCGACAAAGACAGATTC<br>TTTGAGGGAGCTAAGCTCAACGTAGTTCTAACAGTTTTTT<br>AATTAGAGAGCAGATCTCTGATGAATAACCAACGGAAA<br>AAGGCGAGAAACACGCCTTTCAATATGCTGAAACGCGA<br>GAGAAACCGCGTGTCGACTGTGCAACAGCTGACAAAGA<br>GATTCTCACTTGGAATGCTGCAGGGACGAGGACCATTAA<br>AACTGTTCATGGCCCTGGTGGCGTTCCTTCGT*CTCTCTCC*<br>*CTCATAAAGAAAGGCCCGGCCTTTTCCTCT*TAGAAAGCAGA<br>ACTAATTTAAAACAAGGCTGGAAGTCAGGTCGGATTAAG<br>CCATAGTACGGAAAAAACTATGCTACCTGTGAGCCCCGT<br>CCAAGGACGTTAAAAGAAGTCAGGCCATCATAAATGCC<br>ATAGCTTGAGTAAACTATGCAGCCTGTAGCTCCACCTGA<br>GAAGGTGTAAAAAATCCGGGAGGCCACAAACCATGGAA<br>GCTGTACGCATGGCGTAGTGGACTAGCGGTTAGAGGAGA<br>CCCCTCCCTTACAAATCGCAGCAACAATGGGGGCCCAAG<br>GCGAGATGAAGCTGTAGTCTCGCTGGAAGGACTAGAGGT<br>TAGAGGAGACCCCCCCGAAACAAAAAACAGCATATTGA<br>CGCTGGGAAAGACCAGAGATCCTGCTGTCTCCTCAGCAT<br>CATTCCAGGCACAGAACGCCAGAAAATGGAATGGTGCT<br>GTTGAATCAACAGGTTCT|ctagaggatccccgggtaccgagctcgaattcac<br>tggccgtcgt
TBEV 5′-3′ UTR	aagcttgcatgcctgcaggtcgacTAATACGACTCACTATTAGATTTTC<br>TTGCACGTGCATGCGTTTGCTTCGGACAGCATTAGCAGC<br>GGTTGGTTTGAAAGAGATATTCTTTTGTTTCTACCAGTCG<br>TGAACGTGTTGAGAAAAAGACAGCTTAGGAGAACAAGA<br>GCTGGGGATGGTCAAGAAGGCCATCCTGAAAGGTAAGG<br>GGGGCGGTCCCCCTCGACGAGTGTCGAAAGAGACCGCA<br>ACGAAGACGCGTCAACCCAGAGTCCAAATGCCAAATGG<br>GCTTGTGTTGATGCGCATGATGGGGATCTTGTGGCATGC<br>CGTAGCCGGCACCGCGAGAAACCCCGTATT*CTCTCTCCCT*<br>*CATAAAGAAAGGCCCGGCCTTTTCCTCT*TAAACCCAGACTG<br>TGACAGAGCAAAACCCGGAAGGCTCGTAAAAGATTGTC<br>CGGAACCAAAAGAAAAGCAAGCAACTCACAGAGATAGA<br>GCTCGGACTGGAGAGCTCTTTAAACAAAAAAAAAAAAA<br>AAAAAAAAAAAAAAAAAAAAAAAAAAAAAAAAAAG<br>CCAGAATTGAGCTGAACCTGGAGAGCTCATTAAATACAG<br>TCCAGACGAAACAAAACATGACAAAGCAAAGAGGCTGA<br>GCTAAAAGTTCCCACTACGGGACTGCTTCATAGCGGTTT<br>GTGGGGGGAGGCTAGGAGGCGAAGCCACAGATCATGGA

<table>
<tr><td></td><td>ATGATGCGGCAGCGCGCGAGAGCGACGGGGAAGTGGTC
GCACCCGACGCACCATCCATGAAGCAATACTTCGTGAGA
CCCCCCCTGACCAGCAAAGGGGGCAGACCGGTCAGGGG
TGAGGAATGCCCCCAGAGTGCATTACGGCAGCACGCCAG
TGAGAGTGGCGACGGAAAATGGTCGATCCCGACGTAG
GGCACTCTGAAAAATTTTGTGAGACCCCCTGCATCATGA
TAAGGCCGAACATGGTGCATGAAAGGGGAGGCCCCCGG
AAGCACGCTTCCGGGAGGAGGGAAGAGAGAAATTGGCA
GCTCTCTTCAGGATTTTTCCTCCTCCTATACAAAATTCCC
CCTCGGTAGAGGGGGGGCGGTTCTTGTTCTCCCTGAGCC
ACCATCACCCAGACACAGGTAGTCTGACAAGGAGGTGAT
GTGTGACTCGGAAAAACACCCGCT|ctagaggatccccgggtacc</td></tr>
<tr><td>WNV 5′-3′ UTR</td><td>ttgcatgcctgcaggtcgacTAATACGACTCACTATTAGTAGTTCGC
CTGTGTGAGCTGACAAACTTAGTAGTGTTTGTGAGGATT
AACAACAATTAACACAGTGCGAGCTGTTTCTTAGCACGA
AGATCTCGATGTCTAAGAAACCAGGAGGGCCCGGCAAG
AGCCGGGCTGTCAATATGCTAAAACGCGGAATGCCCCGC
GTGTTGTCCTTGATTGGACTGAAGAGGGCTATGTTGAGC
CTGATCGACGGCAAGGGGCCAATACGATTTGTGTTGGCT
CTCTTGGCGTTCTTCAGGTTCACAGCACTCTCTCCCTCATA
AAGAAAGGCCCGGCCTTTTCCTCTTAGATATTTAATCAATT
GTAAATAGACAATATAAGTATGCATAAAAGTGTAGTTTT
ATAGTAGTATTTAGTGGTGTTAGTGTAAATAGTTAAGAA
AATTTTGAGGAGAAAGTCAGGCCGGGAAGTTCCCGCCAC
CGGAAGTTGAGTAGACGGTGCTGCCTGCGACTCAACCCC
AGGAGGACTGGGTGAACAAAGCCGCGAAGTGATCCATG
TAAGCCCTCAGAACCGTCTCGGAAGGAGGACCCCACATG
TTGTAACTTCAAAGCCCAATGTCAGACCACGCTACGGCG
TGCTACTCTGCGGAGAGTGCAGTCTGCGATAGTGCCCCA
GGAGGACTGGGTTAACAAAGGCAAACCAACGCCCCACG
CGGCCCTAGCCCCGGTAATGGTGTTAACCAGGGCGAAAG
GACTAGAGGTTAGAGGAGACCCCGCGGTTTAAAGTGCAC
GGCCCAGCCTGGCTGAAGCTGTAGGTCAGGGGAAGGAC
TAGAGGTTAGTGGAGACCCCGTGCCACAAAACACCACA
ACAAAACAGCATATTGACACCTGGGATAGACTAGGAGA
TCTTCTGCTCTGCACAACCAGCCACACGGCACAGTGCGC
CGACAATGGTGGCTGGTGGTGCGAGAACACAGGATCT|cta
gaggatccccgggtacc</td></tr>
<tr><td>ZIKV 5′-3′ UTR</td><td>tgaccatgattacgccaagcttgcatgcctgcaggtcgacTAATACGACTCACT
ATTAGTTGTTGATCTGTGTGAATCAGACTGCGACAGTTC
GAGTTTGAAGCGAAAGCTAGCAACAGTATCAACAGGTTT
TATTTTGGATTTGGAAACGAGAGTTTCTGGTCATGAAAA
ACCCAAAAAAGAAATCCGGAGGATTCCGGATTGTCAATA
TGCTAAAACGCGGAGTAGCCCGTGTGAGCCCCTTTGGGG
GCTTGAAGAGGCTGCCAGCCGGACTTCTGCTGGGTCATG
GGCCCATCAGGATGGTCTTGGCAATTCTAGCCTTTTTGAG</td></tr>
</table>

<table>
<tr><td></td><td>AT*CTCTCTCCCTCATAAAGAAAGGCCCGGCCTTTTCCTCT*TA
AGCACCAATCTTAATGTTGTCAGGCCTGCTAGTCAGCCA
CAGCTTGGGGAAAGCTGTGCAGCCTGTGACCCCCCCAGG
AGAAGCTGGGAAACCAAGCCTATAGTCAGGCCGAGAAC
GCCATGGCACGGAAGAAGCCATGCTGCCTGTGAGCCCCT
CAGAGGACACTGAGTCAAAAAACCCCACGCGCTTGGAG
GCGCAGGATGGGAAAAGAAGGTGGCGACCTTCCCCACC
CTTCAATCTGGGGCCTGAACTGGAGATCAGCTGTGGATC
TCCAGAAGAGGGACTAGTGGTTAGAGGAGACCCCCCGG
AAAACGCAAAACAGCATATTGACGCTGGGAAAGACCAG
AGACTCCATGAGTTTCCACCACGCTGGCCGCCAGGCACA
GATCGCCGAATAGCGGCGGCCGGTGTGGGGAAATCCAT
GGGTCT|ctagaggatccccgggtaccgagctcgaattcactggccg</td></tr>
<tr><td>DENV2 CS
mutant 1 5′-3′
UTR</td><td>tgaccatgattacgccaagcttgcatgcctgcaggtcgacTAATACGACTCACT
ATAGAGTTGTTAGTCTACGTGGACCGACAAAGACAGATT
CTTTGAGGGAGCTAAGCTCAACGTAGTTCTAACAGTTTTT
TAATTAGAGAGCAGATCTCTGATGAATAACCAACGGAAA
AAGGCGAGAAACACGCCTTTGCTAGTGCATAAACGCGA
GAGAAACCGCGTGTCGACTGTGCAACAGCTGACAAAGA
GATTCTCACTTGGAATGCTGCAGGGACGAGGACCATTAA
AACTGTTCATGGCCCTGGTGGCGTTCCTTCGT*CTCTCTCC*
*CTCATAAAGAAAGGCCCGGCCTTTTCCTCT*TAGAAAGCAGA
ACTAATTTAAAACAAGGCTGGAAGTCAGGTCGGATTAAG
CCATAGTACGGAAAAAACTATGCTACCTGTGAGCCCCGT
CCAAGGACGTTAAAAGAAGTCAGGCCATCATAAATGCC
ATAGCTTGAGTAAACTATGCAGCCTGTAGCTCCACCTGA
GAAGGTGTAAAAAATCCGGGAGGCCACAAACCATGGAA
GCTGTACGCATGGCGTAGTGGACTAGCGGTTAGAGGAGA
CCCCTCCCTTACAAATCGCAGCAACAATGGGGGCCCAAG
GCGAGATGAAGCTGTAGTCTCGCTGGAAGGACTAGAGGT
TAGAGGAGACCCCCCCGAAACAAAAAACAGCATATTGA
CGCTGGGAAAGACCAGAGATCCTGCTGTCTCCTCAGCAT
CATTCCAGGCACAGAACGCCAGAAAATGGAATGGTGCT
GTTGAATCAACAGGTTCT|ctagaggatccccgggtaccgagctcgaattcac
tggccgtcgt</td></tr>
<tr><td>DENV2 CS
rescue 1 5′-3′
UTR</td><td>tgaccatgattacgccaagcttgcatgcctgcaggtcgacTAATACGACTCACT
ATAGAGTTGTTAGTCTACGTGGACCGACAAAGACAGATT
CTTTGAGGGAGCTAAGCTCAACGTAGTTCTAACAGTTTTT
TAATTAGAGAGCAGATCTCTGATGAATAACCAACGGAAA
AAGGCGAGAAACACGCCTTTGCTAGTGCATAAACGCGA
GAGAAACCGCGTGTCGACTGTGCAACAGCTGACAAAGA
GATTCTCACTTGGAATGCTGCAGGGACGAGGACCATTAA
AACTGTTCATGGCCCTGGTGGCGTTCCTTCGT*CTCTCTCC*
*CTCATAAAGAAAGGCCCGGCCTTTTCCTCT*TAGAAAGCAGA
ACTAATTTAAAACAAGGCTGGAAGTCAGGTCGGATTAAG
CCATAGTACGGAAAAAACTATGCTACCTGTGAGCCCCGT</td></tr>
</table>

	CCAAGGACGTTAAAAGAAGTCAGGCCATCATAAATGCC<br>ATAGCTTGAGTAAACTATGCAGCCTGTAGCTCCACCTGA<br>GAAGGTGTAAAAAATCCGGGAGGCCACAAACCATGGAA<br>GCTGTACGCATGGCGTAGTGGACTAGCGGTTAGAGGAGA<br>CCCCTCCCTTACAAATCGCAGCAACAATGGGGGCCCAAG<br>GCGAGATGAAGCTGTAGTCTCGCTGGAAGGACTAGAGGT<br>TAGAGGAGACCCCCCCGAAACAAAAAAATGCACTAGCA<br>CGCTGGGAAAGACCAGAGATCCTGCTGTCTCCTCAGCAT<br>CATTCCAGGCACAGAACGCCAGAAAATGGAATGGTGCT<br>GTTGAATCAACAGGTTCT|ctagaggatccccgggtaccgagctcgaattcac<br>tggccgtcgt
DENV2 CS mutant 2 5′-3′ UTR	tgaccatgattacgccaagcttgcatgcctgcaggtcgacTAATACGACTCACT<br>ATAGAGTTGTTAGTCTACGTGGACCGACAAAGACAGATT<br>CTTTGAGGGAGCTAAGCTCAACGTAGTTCTAACAGTTTTT<br>TAATTAGAGAGCAGATCTCTGATGAATAACCAACGGAAA<br>AAGGCGAGAAACACGCCTTTCAATATGCTGAAACGCGA<br>GAGAAACCGCGTGTCGACTGTGCAACAGCTGACAAAGA<br>GATTCTCACTTGGAATGCTGCAGGGACGAGGACCATTAA<br>AACTGTTCATGGCCCTGGTGGCGTTCCTTCGT*CTCTCTCC*<br>*CTCATAAAGAAAGGCCCGGCCTTTTCCTCT*TAGAAAGCAGA<br>ACTAATTTAAAACAAGGCTGGAAGTCAGGTCGGATTAAG<br>CCATAGTACGGAAAAAACTATGCTACCTGTGAGCCCCGT<br>CCAAGGACGTTAAAAGAAGTCAGGCCATCATAAATGCC<br>ATAGCTTGAGTAAACTATGCAGCCTGTAGCTCCACCTGA<br>GAAGGTGTAAAAAATCCGGGAGGCCACAAACCATGGAA<br>GCTGTACGCATGGCGTAGTGGACTAGCGGTTAGAGGAGA<br>CCCCTCCCTTACAAATCGCAGCAACAATGGGGGCCCAAG<br>GCGAGATGAAGCTGTAGTCTCGCTGGAAGGACTAGAGGT<br>TAGAGGAGACCCCCCCGAAACAAAAAAAATGACACTCA<br>CGCTGGGAAAGACCAGAGATCCTGCTGTCTCCTCAGCAT<br>CATTCCAGGCACAGAACGCCAGAAAATGGAATGGTGCT<br>GTTGAATCAACAGGTTCT|ctagaggatccccgggtaccgagctcgaattcac<br>tggccgtcgt
DENV2 CS rescue 2 5′-3′ UTR	tgaccatgattacgccaagcttgcatgcctgcaggtcgacTAATACGACTCACT<br>ATAGAGTTGTTAGTCTACGTGGACCGACAAAGACAGATT<br>CTTTGAGGGAGCTAAGCTCAACGTAGTTCTAACAGTTTTT<br>TAATTAGAGAGCAGATCTCTGATGAATAACCAACGGAAA<br>AAGGCGAGAAACACGCCTTTGAGTGTCATTAAACGCGAG<br>AGAAACCGCGTGTCGACTGTGCAACAGCTGACAAAGAG<br>ATTCTCACTTGGAATGCTGCAGGGACGAGGACCATTAAA<br>ACTGTTCATGGCCCTGGTGGCGTTCCTTCGT*CTCTCTCCCT*<br>*CATAAAGAAAGGCCCGGCCTTTTCCTCT*TAGAAAGCAGAAC<br>TAATTTAAAACAAGGCTGGAAGTCAGGTCGGATTAAGCC<br>ATAGTACGGAAAAAACTATGCTACCTGTGAGCCCCGTCC<br>AAGGACGTTAAAAGAAGTCAGGCCATCATAAATGCCAT<br>AGCTTGAGTAAACTATGCAGCCTGTAGCTCCACCTGAGA

	AGGTGTAAAAAATCCGGGAGGCCACAAACCATGGAAGCTGTACGCATGGCGTAGTGGACTAGCGGTTAGAGGAGACCCCTCCCTTACAAATCGCAGCAACAATGGGGGCCCAAGGCGAGATGAAGCTGTAGTCTCGCTGGAAGGACTAGAGGTTAGAGGAGACCCCCCCGAAACAAAAAAAATGACACTCACGCTGGGAAAGACCAGAGATCCTGCTGTCTCCTCAGCATCATTCCAGGCACAGAACGCCAGAAAATGGAATGGTGCTGTTGAATCAACAGGTTCT|ctagaggatccccgggtaccgagctcgaattcactggccgtcgt
DENV2 UAR mutant 1 5′-3′ UTR	tgaccatgattacgccaagcttgcatgcctgcaggtcgacTAATACGACTCACTATAGAGTTGTTAGTCTACGTGGACCGACAAAGACAGATTCTTTGAGGGAGCTAAGCTCAACGTAGTTCTAACAGTTTTTTAATTAGAGACGTCATCTCTGATGAATAACCAACGGAAAAAGGCGAGAAACACGCCTTTCAATATGCTGAAACGCGAGAGAAACCGCGTGTCGACTGTGCAACAGCTGACAAAGAGATTCTCACTTGGAATGCTGCAGGGACGAGGACCATTAAAACTGTTCATGGCCCTGGTGGCGTTCCTTCGT*CTCTCTCCCTCATAAAGAAAGGCCCGGCCTTTTCCTCT*TAGAAAGCAGAACTAATTTAAAACAAGGCTGGAAGTCAGGTCGGATTAAGCCATAGTACGGAAAAAACTATGCTACCTGTGAGCCCCGTCCAAGGACGTTAAAAGAAGTCAGGCCATCATAAATGCCATAGCTTGAGTAAACTATGCAGCCTGTAGCTCCACCTGAGAAGGTGTAAAAAATCCGGGAGGCCACAAACCATGGAAGCTGTACGCATGGCGTAGTGGACTAGCGGTTAGAGGAGACCCCTCCCTTACAAATCGCAGCAACAATGGGGGCCCAAGGCGAGATGAAGCTGTAGTCTCGCTGGAAGGACTAGAGGTTAGAGGAGACCCCCCCGAAACAAAAAACAGCATATTGACGCTGGGAAAGACCAGAGATCCTGCTGTCTCCTCAGCATCATTCCAGGCACAGAACGCCAGAAAATGGAATGGTGCTGTTGAATCAACAGGTTCT|ctagaggatccccgggtaccgagctcgaattcactggccgtcgt
DENV2 UAR mutant 2 5′-3′ UTR	tgaccatgattacgccaagcttgcatgcctgcaggtcgacTAATACGACTCACTATAGAGTTGTTAGTCTACGTGGACCGACAAAGACAGATTCTTTGAGGGAGCTAAGCTCAACGTAGTTCTAACAGTTTTTTAATTACAGAGCACATCTGTGATGAATAACCAACGGAAAAAGGCGAGAAACACGCCTTTCAATATGCTGAAACGCGAGAGAAACCGCGTGTCGACTGTGCAACAGCTGACAAAGAGATTCTCACTTGGAATGCTGCAGGGACGAGGACCATTAAAACTGTTCATGGCCCTGGTGGCGTTCCTTCGT*CTCTCTCCCTCATAAAGAAAGGCCCGGCCTTTTCCTCT*TAGAAAGCAGAACTAATTTAAAACAAGGCTGGAAGTCAGGTCGGATTAAGCCATAGTACGGAAAAAACTATGCTACCTGTGAGCCCCGTCCAAGGACGTTAAAAGAAGTCAGGCCATCATAAATGCCATAGCTTGAGTAAACTATGCAGCCTGTAGCTCCACCTGAGAAGGTGTAAAAAATCCGGGAGGCCACAAACCATGGAAGCTGTACGCATGGCGTAGTGGACTAGCGGTTAGAGGAGA

<table>
<tr><td></td><td>CCCCTCCCTTACAAATCGCAGCAACAATGGGGGCCCAAGGCGAGATGAAGCTGTAGTCTCGCTGGAAGGACTAGAGGTTAGAGGAGACCCCCCCGAAACAAAAAACAGCATATTGACGCTGGGAAAGACCAGAGATCCTGCTGTCTCCTCAGCATCATTCCAGGCACAGAACGCCAGAAAATGGAATGGTGCTGTTGAATCAACAGGTTCT|ctagaggatccccgggtaccgagctcgaattcactggccgtcgt</td></tr>
<tr><td>DENV1 5′-3′ UTR</td><td>tgaccatgattacgccaagcttgcatgcctgcaggtcgacTAATACGACTCACTATTAGTTGTTAGTCTACGTGGACCGACAAGAACAGTTTCGAATCGGAAGCTTGCTTAACGTAGTTCTAACAGTTTTTTATTAGAGAGCGGATCTCTGATGAACAACCAACGGAAAAAGACGGGTCGACCGTCTTTCAATATGCTGAAACGCGCGAGAAACCGCGTGTCAACTGGTTCACAGTTGGCGAAGAGATTCTCAAAAGGATTGCTTTCAGGCCAAGGACCCATGAAATTGGTGATGGCTTTCATAGCATTTCTAAGAT*CTCTCTCCCTCATAAAGAAAGGCCCGGCCTTTTCCTCT*TAAGTCAACACACTCATGAAACAAAGGAAAATAGAAGACCAAACAAAGTAAGAAGTCAGGCCAGATTAAGCCATAGTACGGAAAAAGCTATGCTGCCTGTGAGCCCCGTCCAAGGACGTAAAATGAAGTCAGGCCGAAAGCCACGGATTGAGCAAGCCGTGCTGCCTGTGGCTCCATCGTGGGGATGTAAAAACCCGGGAGGCTGCAACCCATGGAAGCTGTACGCATGGGGTAGCAGACTAGTGGTTAGAGGAGACCCCTCCCTAGACATAACGCAGCAGCGGGGCCCAACACCAGGGGAAGCTGTACCTTGGTGGTAAGGACTAGAGGTTAGAGGAGACCCCCCGCACAACAACAAACAGCATATTGACGCTGGGAGAGACCAGAGATCCTGCTGTCTCTACAGCATCATTCCAGGCACAGAACGCCAGAAAATGGAATGGTGCTGTTGAATCAACAGGTTCT|ctagaggatccccgggtaccgagctcgaattcactggccg</td></tr>
<tr><td>DENV3 5′-3′ UTR</td><td>tgaccatgattacgccaagcttgcatgcctgcaggtcgacTAATACGACTCACTATTAGTTGTTAGTCTACGTGGACCGACAAGAACAGTTTCGACTCGGAAGCTTGCTTAACGTAGTGCTGACAGTTTTTTATTAGAGAGCAGATCTCTGATGAACAACCAACGGAAGAAGACGGGAAAACCGTCTATCAATATGCTGAAACGCGTGAGAAACCGTGTGTCAACTGGATCACAGTTGGCGAAGAGATTCTCAAAAGGACTGCTGAACGGCCAGGGACCAATGAAATTGGTTATGGCGTTCATAGCTTTCCTCAGAT*CTCTCTCCCTCATAAAGAAAGGCCCGGCCTTTTCCTCT*TAAACGTAGGAAGTGGAAAAGAGGCTAACTGTCAGGCCACCTTAAGCCACAGTACGGAAGAAGCTGTGCTGCCTGTGAGCCCCGTCCAAGGACGTTAAAAGAAGAAGTCAGGCCCCAAAGCCACGGTTTGAGCAAACCGTGCTGCCTGTAGCTCCGTCGTGGGGACGTAAAACCTGGGAGGCTGCAAACTGTGGAAGCTGTACGCACGGTGTAGCAGACTAGCGGTTAGAGGAGACCCCTCCCATGACACAACGCAGCAGCGGGGCCCGAGCACTGAGGGAAGCTGTACCTCCTTGCAAAGGACTAGAGGTTAGAGGAGACCC</td></tr>
</table>

<table>
<tr><td></td><td>CCCGCAAATAAAAACAGCATATTGACGCTGGGAGAGAC
CAGAGATCCTGCTGTCTCCTCAGCATCATTCCAGGCACA
GAACGCCAGAAAATGGAATGGTGCTGTTGAATCAACAG
GTTCT|ctagaggatccccgggtaccgagctcgaattcactggccg</td></tr>
<tr><td>DENV4 5′-3′
UTR</td><td>tgaccatgattacgccaagcttgcatgcctgcaggtcgacTAATACGACTCACT
ATTAGTTGTTAGTCTGTGTGGACCGACAAGGACAGTTCC
AAATCGGAAGCTTGCTTAACACAGTTCTAACAGTTTGTTT
GAATAGAGAGCAGATCTCTGGAAAAATGAACCAACGAA
AAAAGGTGGTTAGACCACCTTTCAATATGCTGAAACGCG
AGAGAAACCGCGTATCAACCCCTCAAGGGTTGGTGAAG
AGATTCTCAACCGGACTTTTTTCTGGGAAAGGACCCTTA
CGGATGGTGCTAGCATTCATCACGTTTTTGCGAGTCCTTT
CCATCCCCTCTCTCCCTCATAAAGAAAGGCCCGGCCTTTTCC
TCTTAATTACCAACAACAAACACCAAAGGCTATTGAAGT
CAGGCCACTTGTGCCACGGTTTGAGCAAACCGTGCTGCC
TGTAGCTCCGCCAATAATGGGAGGCGTAATAATCCCCAG
GGAGGCCATGCGCCACGGAAGCTGTACGCGTGGCATATT
GGACTAGCGGTTAGAGGAGACCCCTCCCATCACTGACAA
AACGCAGCAAAAGGGGGCCCGAAGCCAGGAGGAAGCTG
TACTCCTGGTGGAAGGACTAGAGGTTAGAGGAGACCCCC
CCAACACAAAAACAGCATATTGACGCTGGGAAAGACCA
GAGATCCTGCTGTCTCTGCAACATCAATCCAGGCACAGA
GCGCCGCAAGATGGATTGGTGTTGTTGATCCAACAGGTT
CT|ctagaggatccccgggtaccgagctcgaattcactggccg</td></tr>
<tr><td>DENV1 SLB/3′-
SL mutant 5′-3′
UTR</td><td>tgaccatgattacgccaagcttgcatgcctgcaggtcgacTAATACGACTCACT
ATTAGTTGTTAGTCTACGTGGACCGACAAGAACAGTTTC
GAATCGGAAGCTTGCTTAACGTAGTTCTAACAGTTTTTTA
ATTAGAGAGCAGATCTCTGATGAATAACCAACGGAAAA
AGACGGGTCGACCGTCTTTCAATATGCTGAAACGCGCGA
GAAACCGCGTGTCAACTGGTTCACAGTTGGCGAAGAGAT
TCTCAAAAGGATTGCTTTCAGGCCAAGGACCCATGAAAT
TGGTGATGGCTTTCATAGCATTTCTAAGATCTCTCTCCCT
CATAAAGAAAGGCCCGGCCTTTTCCTCTTAAGTCAACACAC
TCATGAAACAAAGGAAAATAGAAGACCAAACAAAGTAA
GAAGTCAGGCCAGATTAAGCCATAGTACGGAAAAAGCT
ATGCTGCCTGTGAGCCCCGTCCAAGGACGTAAAATGAAG
TCAGGCCGAAAGCCACGGATTGAGCAAGCCGTGCTGCCT
GTGGCTCCATCGTGGGGATGTAAAAACCCGGGAGGCTGC
AACCCATGGAAGCTGTACGCATGGGGTAGCAGACTAGTG
GTTAGAGGAGACCCCTCCCTAGACATAACGCAGCAGCGG
GGCCCAACACCAGGGGAAGCTGTACCTTGGTGGTAAGG
ACTAGAGGTTAGAGGAGACCCCCCGCACAACAACAAAC
AGCATATTGACGCTGGGAGAGACCAGAGATCCTGCTGTC
TCCTCAGCATCATTCCAGGCACAGAACGCCAGAAAATGG
AATGGTGCTGTTGAATCAACAGGTTCT|ctagaggatccccgggtac
cgagctcgaattcactggccg</td></tr>
</table>

<table>
<tr><td>DENV2 SLB/3′-SL mutant 5′-3′ UTR</td><td>tgaccatgattacgccaagcttgcatgcctgcaggtcgacTAATACGACTCACT
ATTAGTTGTTAGTCTACGTGGACCGACAAAGACAGATTC
TTTGAGGGAGCTAAGCTCAACGTAGTTCTAACAGTTTTTT
ATTAGAGAGCGGATCTCTGATGAACAACCAACGGAAAA
AGGCGAGAAACACGCCTTTCAATATGCTGAAACGCGAG
AGAAACCGCGTGTCGACTGTGCAACAGCTGACAAAGAG
ATTCTCACTTGGAATGCTGCAGGGACGAGGACCATTAAA
ACTGTTCATGGCCCTGGTGGCGTTCCTTCGT*CTCTCTCCCT*
*CATAAAGAAAGGCCCGGCCTTTTCCTCT*TAGAAAGCAGAAC
TAATTTAAAACAAGGCTGGAAGTCAGGTCGGATTAAGCC
ATAGTACGGAAAAAACTATGCTACCTGTGAGCCCCGTCC
AAGGACGTTAAAAGAAGTCAGGCCATCATAAATGCCAT
AGCTTGAGTAAACTATGCAGCCTGTAGCTCCACCTGAGA
AGGTGTAAAAAATCCGGGAGGCCACAAACCATGGAAGC
TGTACGCATGGCGTAGTGGACTAGCGGTTAGAGGAGACC
CCTCCCTTACAAATCGCAGCAACAATGGGGGCCCAAGGC
GAGATGAAGCTGTAGTCTCGCTGGAAGGACTAGAGGTTA
GAGGAGACCCCCCCGAAACAAAAAACAGCATATTGACG
CTGGGAAAGACCAGAGATCCTGCTGTCTCTACAGCATCA
TTCCAGGCACAGAACGCCAGAAAATGGAATGGTGCTGTT
GAATCAACAGGTTCT|ctagaggatccccgggtaccgagctcgaattcactggc
cg</td></tr>
<tr><td>DENV2 5′ UTR-NanoLuc-3′ UTR</td><td>tgaccatgattacgccaagcttgcatgcctgcaggtcgacTAATACGACTCACT
ATTAGTTGTTAGTCTACGTGGACCGACAAAGACAGATTC
TTTGAGGGAGCTAAGCTCAACGTAGTTCTAACAGTTTTTT
AATTAGAGAGCAGATCTCTGATGAATAACCAACGGAAA
AAGGCGAGAAACACGCCTTTCAATATGCTGAAACGCGA
GAGAAACCGCGTGTCGACTGTGCAACAGCTGACAAAGA
GATTCTCACTTGGAATGCTGCAGGGACGAGGACCATTAA
AACTGTTCATGGCCCTGGTGGCGTTCCTTCGTATGGTCTT
CACACTCGAAGATTTCGTTGGGGACTGGCGACAGACAGC
CGGCTACAACCTGGACCAAGTCCTTGAACAGGGAGGTGT
GTCCAGTTTGTTTCAGAATCTCGGGGTGTCCGTAACTCCG
ATCCAAAGGATTGTCCTGAGCGGTGAAAATGGGCTGAAG
ATCGACATCCATGTCATCATCCCGTATGAAGGTCTGAGC
GGCGACCAAATGGGCCAGATCGAAAAAATTTTTAAGGTG
GTGTACCCTGTGGATGATCATCACTTTAAGGTGATCCTGC
ACTATGGCACACTGGTAATCGACGGGGTTACGCCGAACA
TGATCGACTATTTCGGACGGCCGTATGAAGGCATCGCCG
TGTTCGACGGCAAAAAGATCACTGTAACAGGGACCCTGT
GGAACGGCAACAAAATTATCGACGAGCGCCTGATCAAC
CCCGACGGCTCCCTGCTGTTCCGAGTAACCATCAACGGA
GTGACCGGCTGGCGGCTGTGCGAACGCATTCTGGCGTAG
AAAGCAGAACTAATTTAAAACAAGGCTGGAAGTCAGGT
CGGATTAAGCCATAGTACGGAAAAAACTATGCTACCTGT
GAGCCCCGTCCAAGGACGTTAAAAGAAGTCAGGCCATC</td></tr>
</table>

<table>
<tr><td></td><td>ATAAATGCCATAGCTTGAGTAAACTATGCAGCCTGTAGC
TCCACCTGAGAAGGTGTAAAAAATCCGGGAGGCCACAA
ACCATGGAAGCTGTACGCATGGCGTAGTGGACTAGCGGT
TAGAGGAGACCCCTCCCTTACAAATCGCAGCAACAATGG
GGGCCCAAGGCGAGATGAAGCTGTAGTCTCGCTGGAAG
GACTAGAGGTTAGAGGAGACCCCCCCGAAACAAAAAAC
AGCATATTGACGCTGGGAAAGACCAGAGATCCTGCTGTC
TCCTCAGCATCATTCCAGGCACAGAACGCCAGAAAATGG
AATGGTGCTGTTGAATCAACAGGTTCT|ctagaggatccccgggtac
cgagctcgaattcactggccgtcgt</td></tr>
</table>

Table S3. E_{FRET} distribution fits. Excel datasheet containing results Gaussian distribution fitting to cumulative E_{FRET} distributions. Mean E_{FRET} (μ), standard deviation (σ), and normalized amplitude (a) with 95% confidence intervals of the fit are reported for each individual peak fit by the cftool package in MATLAB.
